# Plasmon–Exciton Coupling Effect in Nanostructured Arrays for Optical Signal Amplification and SARS-CoV-2 DNA Sensing

**DOI:** 10.1021/acsanm.2c05063

**Published:** 2023-01-24

**Authors:** Frank Tukur, Bhawna Bagra, Anitha Jayapalan, Mengxin Liu, Panesun Tukur, Jianjun Wei

**Affiliations:** Department of Nanoscience, Joint school of Nanoscience and Nanoengineering, University of North Carolina at Greensboro, Greensboro, North Carolina27401, United States

**Keywords:** nanoslit array, acridine, plasmon−exciton coupling, signal amplification, DNA sensing, SARS-CoV-2

## Abstract

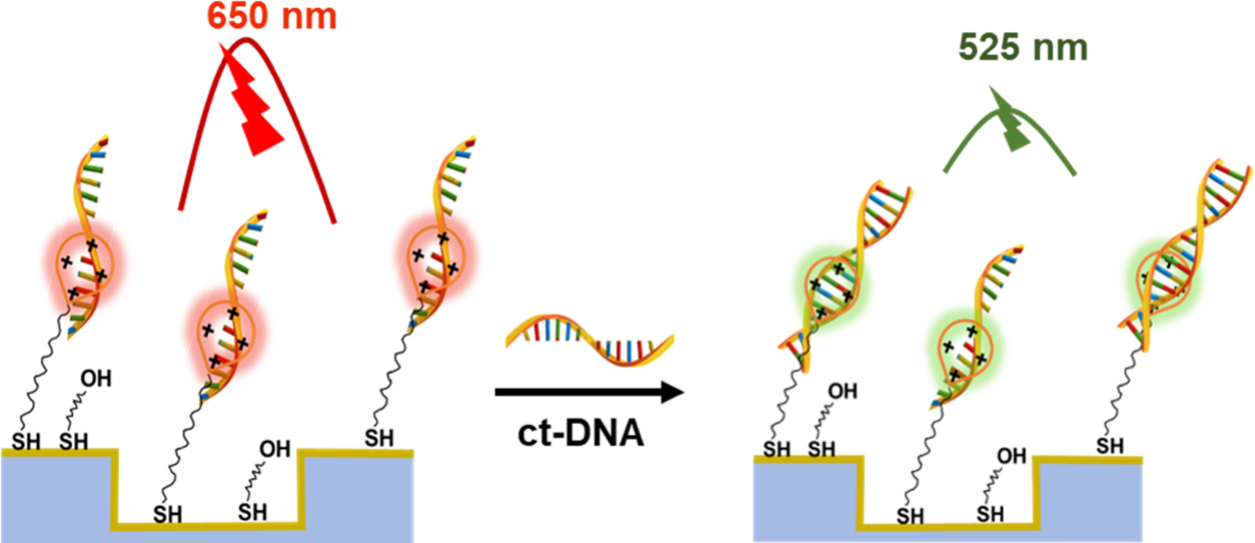

A surface plasmon resonance (SPR)-enhanced optical signal using a nanoslit array and acridine orange (AO) dye system at a flexible poly(dimethylsiloxane) (PDMS) substrate was achieved in this work and demonstrated a simple sensing scheme to directly detect SARS-CoV-2 nucleic acid via DNA hybridization. A simple nanoimprinting pattern transfer technique was introduced to form uniform reproducible nanoslit arrays where the dimensions of the slit array were controlled by the thickness of the gold film. The plasmon–exciton coupling effect on the optical enhancement of different dye molecules, i.e., AO, propidium iodide (PI), or dihydroethidium (DHE) attached to the nanoslit surfaces, was examined thoroughly by measuring the surface reflection and fluorescence imaging. The results indicate that the best overlap of the plasmon resonance wavelength to the excitation spectrum of AO presented the largest optical enhancement (∼57×) compared to the signal at flat gold surfaces. Based on this finding, a sensitive assay for detecting DNA hybridization was generated using the interaction of the selected SARS-CoV-2 ssDNA and dsDNA with AO to trigger the metachromatic behavior of the dye at the nanoarray surfaces. We found strong optical signal amplification on the formation of acridine-ssDNA complexes and a quenched signal upon hybridization to the complementary target DNA (ct-DNA) along with a blue shift in the fluorescence of AO-dsDNAs. A quantitative evaluation of the ct-DNA concentration in a range of 100–0.08 nM using both the reflection and emission imaging signals demonstrated two linear regimes with a lowest detection limit of 0.21 nM. The sensing method showed high sensitivity and distinguished signals from 1-, 2-, and 3-base mismatched DNA targets, as well as high stability and reusability. This approach toward enhancing optical signal for DNA sensing offers promise in a general, rapid, and direct vision detection method for nucleic acid analytes.

## Introduction

While tremendous progress has been made in the development of plasmon-enhanced fluorescence (PEF) of emitters placed in the vicinity of a metal-dielectric nanostructure (NS) interface, there still exists a growing need for NS designs that offer high optical signal for portable and wearable point-of-care analytical devices. Currently, PEF emission systems have demonstrated a practicable application for the detection of biological molecules.^1,2^ Their efficiency in detecting low molecular concentrations depends greatly on the ability of the nanostructures to trap the electromagnetic field that couples well with the surrounding emitter species.^3−5^ This presents an interesting challenge since small variations in the geometry, size, and type of metals used can cause a significant difference in the magnitude and wavelength position of electromagnetic fields concentrated in the NS area. Therefore, a rational design of the plasmonic NS and cautious selection of an emitter whose optoelectronic property is a match for interaction with plasmonic fields is necessary.^6−8^

The interaction of light with an optically active species of nanoscale or less dimension is generally less efficient due to a size mismatch between the species and wavelength of light, which leads to poor energy conversion. To maximize light absorption, emission, or scattering, plasmonic nanostructures have been utilized to confine the electromagnetic field and to strongly couple it with optically active materials.^9−11^ One of the resultant plasmon–exciton coupling systems serves to increase the local density of optical states and consequently improve the spontaneous emission rate commonly referred to as the Purcell effect.^12,13^ This approach has been implemented especially for the significant enhancement of photoluminescence (PL) of quantum dots, carbon nanodots, and molecular dyes placed in the near-field vicinity of a plasmonic NS.^14−17^ Such enhancement offers huge potential applications for increasing the optical absorption and the fluorescence intensity of metal-coupled fluorophores.^13,18^

However, the PL emission can be a cumulative influence of the energy interplay between the metal and the fluorophore in a PEC system. This interaction can lead to either emission enhancement via plasmon-induced resonance energy transfer or quenching via Forster resonance energy transfer (FRET).^19,20^ Such effects depend on whether an emitter is directly attached to a single nanoparticle, planar metallic surface, or placed in a metallic cavity.^20^ Although the absorption of an emitter is enhanced by the near field of a plasmonic NS, attaching it directly to a metal surface may result in fluorescence quenching of the excited state rather than enhancement.^12,21^ This behavior has been explained in terms of the transfer of excited electrons from the emitter (fluorophore) to the metal’s empty orbital.^22^ To mitigate quenching effects, localized high-density electromagnetic field states is required to surround the emitter thereby increasing the number of radiative states. To achieve this, various shapes and configurations that utilize nanoparticle dimers,^12^ nanorods,^23^ nanoholes,^24^ nanoslits,^25^ and bowtie dimers^8^ have been explored. Notably, when an emitter is sandwiched between two or more metal structures, fluorescence emission is enhanced due to strong local field enhancement caused by plasmon–plasmon coupling between adjacent metal structures.^26,27^ This gives nanodimers and nanoslits an edge in practical application over single nanoparticles, nanorods, and flat metallic surfaces. However, careful optimization of the proximity between dimers and nanocavity width is necessary to prevent electron tunneling, which can also result in quenching.^28^

Although the localized surface plasmon resonance (LSPR) excited in nanoparticle dimers offer an enhanced field intensity by many orders of magnitude,^29^ controlling their precise orientation and separation is a challenge.^30,31^ On the other hand, surface plasmon polariton (SPP)-based nanostructures generate a relatively more uniform electric field but suffer from low-intensity field generation. To compensate for each other’s demerits, it is desirable to excite both LSP and SPP modes in a single system. Such an approach may produce much higher field confinement and better PL enhancement.

In this work, we investigate the light–matter interaction between small-molecular fluorescence dyes and a Au nanoslit array structure fabricated on a flexible substrate. The suitability of the Au nanoslit cavity on the substrate toward PL enhancement of three different dye species, namely, acridine orange (AO), propidium iodide (PI), or dihydroethidium (DHE), was examined through the coupling of the species to the plasmonic slit structure. The nanoslit offers one of the best ways of manipulating optical field generation and propagation that gives a promising enhancement of fluorescence intensity due to its ability to simultaneously shelter both LSP resonance (at the top and bottom edges) and SPP (at the two side walls of the slit) modes, providing a superior platform for light–matter interactions where both the emission and excitation of nearby optically active species can be influenced.^5,32−34^ Another advantage of using the nanoslit array lies in its geometry, which allows convenient placement and accommodation of optically active materials and creates avenues toward its widescale adoption in flow-through devices for biomolecular sensing application.^34^ We also investigate the interrelationship of NS-generated plasmon with emission and excitation components of the photoactive species. Our efforts are directed toward determining the excitation/emission wavelength requirement of an emitter necessary for optimum coupling with the plasmonic fields. To achieve this, we studied the optical response of fluorescence molecules (AO, PI, and DHE) with different emission wavelengths varying from ∼422 to 650 nm placed in a gold nanoslit of different widths.

Recently, the world was hit hard by a viral pandemic, COVID-19, which as on October 14, 2022, the WHO reported 620,878,405 confirmed cases including 6,543,138 deaths and still counting. To minimize the harm from this pandemic and better prepare for a future repeat of COVID-19 and other pandemics, rapid and portable diagnostic tools are urgently needed. Currently, viral detection employs methods that require polymerase chain reaction, virus isolation, and enzyme-linked immunosorbent assay (ELISA).^35^ The need for newer methodologies for diagnosis of rapid turnaround time, simple operation, and direct readout continues to rise. Plasmonic-based sensors have displayed high detection speed, sensitivity, and portability and thus promising for disease diagnosis.^36,37^ For instance, combining LSPR of different NPs to optical fibers has demonstrated the advantages of miniaturization and portability in the detection of both small and large biomolecules.^38,39^ Viral biosensors can be categorized based on their viral targets into antigen-, cell-, immune-, and DNA-based sensors.^40−44^ The predictable and specific hybridization of the complementary bases that are based on nucleic acid hybridization of DNA-viral targets have demonstrated preserved reactivity, stability, accessibility, and low cost.

Herein, we further potentiate the feasibility and high efficiency of the AO-based optical enhancement platform for the sensitive detection of COVID-19 nucleic acids. AO is a versatile molecule widely adopted for cell differentiation, staining, and routine quantification of DNA^45^ and bacteria^46^ due to its fluorescence and cationic features. Owing to its optical and electronic properties, it is considered one of the most potent candidates for the development of DNA-targeted drugs and chemotherapy.^47^ Being cationic makes AO suitable for electrostatic interactions with ssDNA and also intercalates with dsDNA.^48,49^ These interactions produce unique changes in the emission intensity and color of the dye molecule. Unlike most dyes that produce identical emission spectra for ssDNA and dsDNA, AO exhibits a metachromatic behavior by distinguishably producing red (650 nm) fluorescence when coupled with ssDNA and green (525 nm) with dsDNA.^50^ This behavior is key in generating a new form of optical behavior that can be used to discriminate between ssDNA and dsDNA. To develop an AO-based surface optical sensor for DNA differentiation, it is necessary to determine the ratio of AO to DNA since certain concentration levels of AO can lead to precipitation or DNA denaturation.^51,52^ Complying with this prerequisite in our nanoslit array system, we adopted a well-tested concentration of AO that circumvents precipitation and denaturation concerns over a very wide range of DNA concentration.^50^

**Figure 1:**
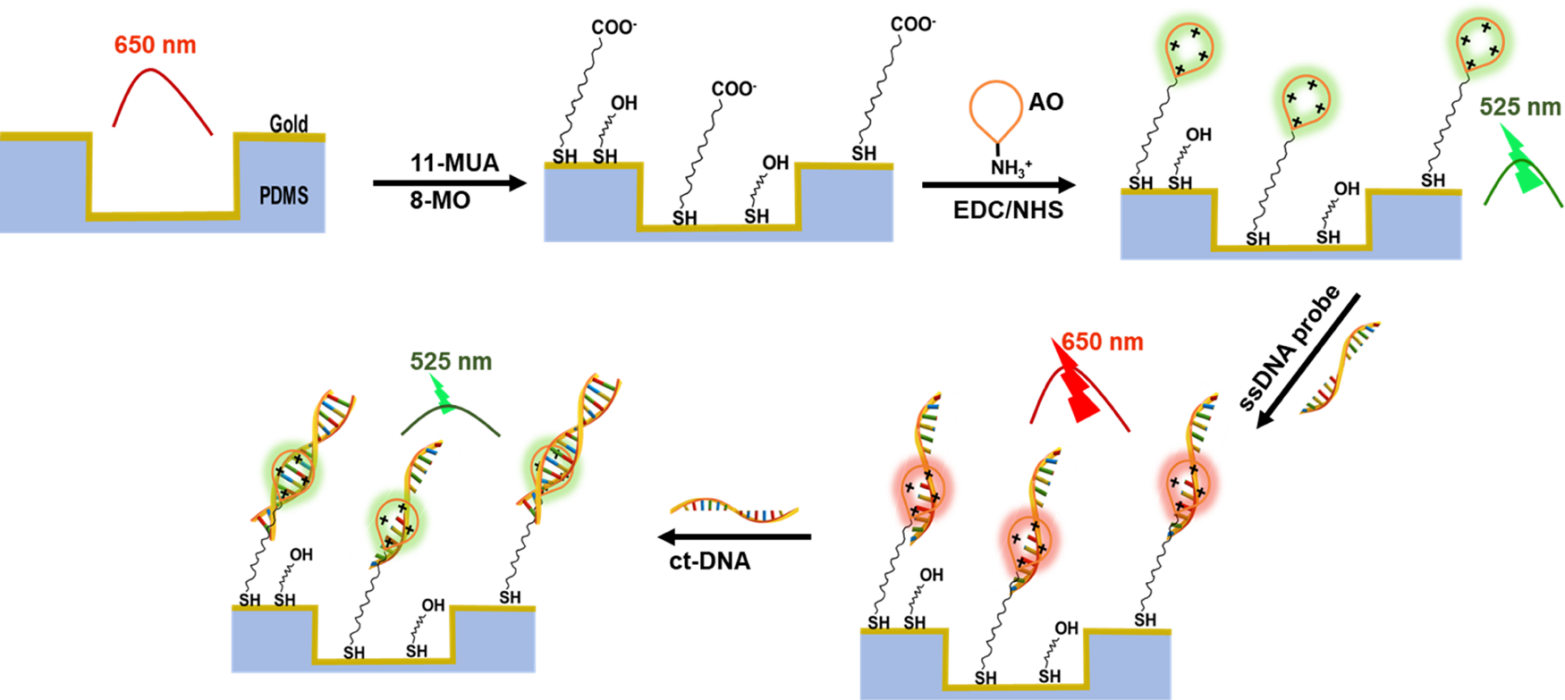
Schematic illustration for the preparation of an optical DNA hybridization sensor and the operational principle.

Figure 1 shows the schematic illustration for the designed optical DNA hybridization sensor and operation principle. The blank Au nanoslit is designed to have a plasmonic resonance peak at around 650 nm. A self-assembled monolayer (SAM) of 11-mercaptoundecanoic acid (11-MUA) and 8-mercaptooctanol (8-MO) at the nanoslit gold surface allows the covalent attachment of AO at the emission of 525 nm. The surface-bound AO could electrostatically interact with ssDNA probes causing AO to shift emission to 650 nm, which matches the best to the plasmonic resonance wavelength, resulting in the enhancement of the optical signal. Finally, the hybridization of the ssDNAs with the ct-DNA species results in a significant decrease of the optical signal at 650 nm because the AO-dsDNA complex emits at 525 nm. The optical response difference is used as signal transduction for detecting DNA hybridization.

## Results and Discussion

### Reversal Nanoimprint to Produce Nanoslit Array Structures

**Figure 2:**
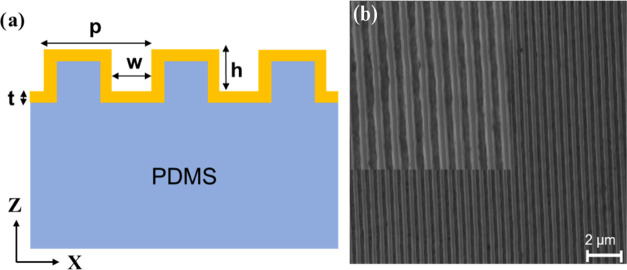
(a) Schematic illustration of the nanoslit array design (not in real geometric ratio) and (b) a representative SEM of a gold film nanoslit array fabricated on a PDMS slab.

Electron beam lithography (EBL) is one of the go-to techniques because it provides highly precise structures in nanoscale dimensions. However, being a direct-writing technique makes it a slow and time-consuming process that is not suitable for mass fabrication. The nanoimprinting technique using a PDMS replication process proved to be a simple and easy control process for low-cost and volume generation of reproducible nanostructured arrays.^53^ The reversal nanoimprint method in this report was optimized to obtain a uniform and reproducible nanoslit array structure (see Scheme S1a). In the process, PDMS was cured on the surface of a Si substrate containing an array of nanoslit structures created using a patterned Si template to produce a negative NS on the PDMS. After pouring PDMS atop Si nanostructures, the curing temperature was maintained at an optimal temperature of 75 °C for 1 h to provide a smooth and reproducible transfer of the nanoslit array to the PDMS. The limitations of this method include challenges associated with the high viscosity of PDMS that makes it difficult to diffuse into smaller nanoscale patterns fabricated on the template. The patterns may thus suffer from low aspect ratios and deformations at different temperatures (Scheme S1b). The deformed NS transfer could occur at a higher baking temperature of 100 °C after 1 h because the PDMS solution solidifies too quickly without giving sufficient time for the solution to flow into the nanoslit. Under 55 °C, peeling the PDMS off the Si wafer is difficult because the PDMS is not fully cured and easily breaks off. Figure 2 shows the design of the nanoslit array and an SEM image of a uniformly imprinted nanoslit array structure. The nanoslits are ∼70 nm wide (*w*), with 300 nm pitch (*p*) and 150 nm deep (*h*), and a deposited gold film of 35 nm thick measured using a crystal quartz microbalance. By changing the deposited gold film thickness, we obtained a series of nanoslit arrays of a variety of slit widths at 55.38, 73.6, 90.1, 115.2, and 135 nm. The slit width decreases with increasing gold film thickness (Figure S1).

### Optical Properties of Nanoslit Arrays

We use both finite-difference time-domain algorithm (FDTD solutions, Lumerical) simulation and experimental measurements of the light reflection at the nanoslit arrays to investigate the SPR effect on the optical properties. Additionally, a validated method based on semianalytical modeling was used to quantitatively account for the scattering, launching, and propagation of surface plasmon at the NS interfaces. First, the optical responses of the nanoslit without or with a Au-base at the slit bottom were simulated with plain light excitation in the reflection mode for a variety of nanoslit widths (*w*). Figure S2 shows the representative cross-sectional renderings of electric field intensity (|*E*|^2^ = *E_x_*^2^ + *E_z_*^2^) distribution in the *xz* plane. The color bar indicates the magnitude of the *E*-field intensity at various regions of the slit. For nanoslits of widths 70, 90, 110, and 130 nm (corresponding to the Au layer thicknesses of 45, 35, 25, 15, and 5 nm) obtained by the FDTD simulations, the maximum fields recorded at the hot spots is 8.08, 6.97, 6.96, and 5.46 eV, respectively. These “hot spots” are located at the entrance edge and bottom corners of the nanoslit structures. They represent a cumulative effect of incident light coupling with localized surface plasmon modes^54^ as well as from surface plasmon polaritons propagating on the top, bottom, and side walls of the nanoslit, which terminates at the edges of the nanoslit causing the formation of a localized electric field.^55^ The magnitude of these localized fields decreases with an increase in the slit width. The |*E*|-field with a decay length of tens of nanometers located at the top and bottom edges of the nanoslit at the Au/air interface indicates the excitation of LSPR. On the other hand, an SPP mode with a longer decay length at the Au/PDMS interface is also visible. The field distribution evidently reveals the coexistence of both SPP and LSPR modes at these “hot spot” regions.^34^

**Figure 3:**
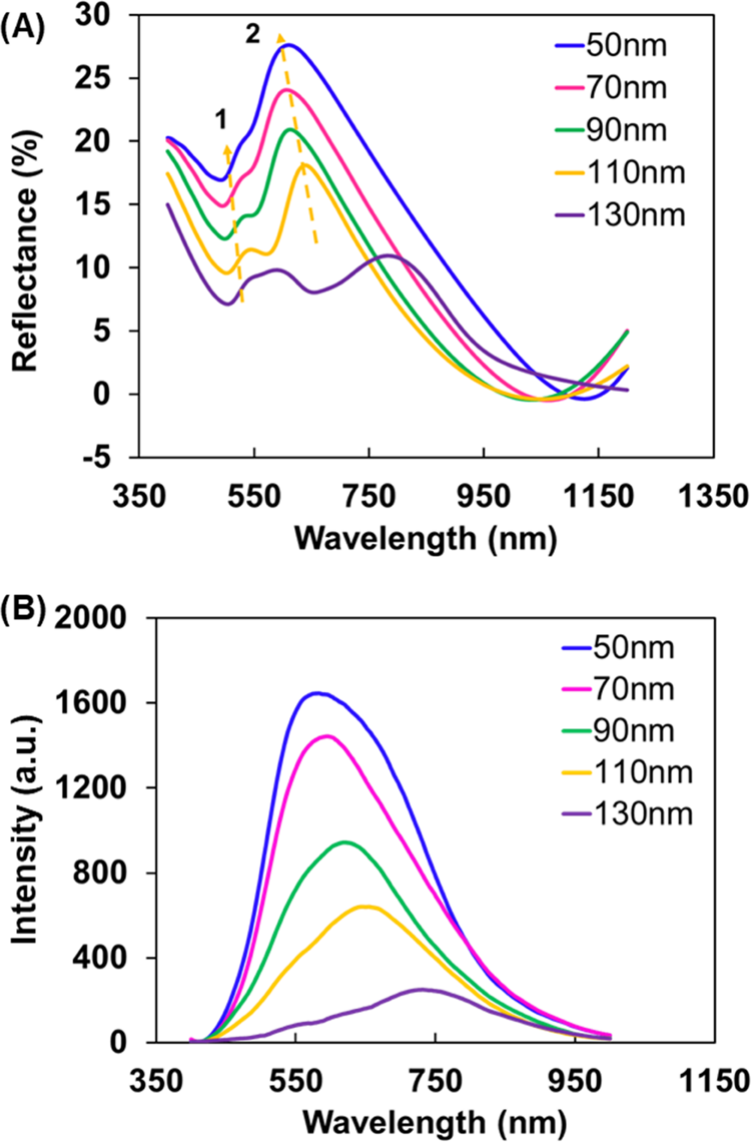
(A) FDTD calculated reflectance spectra of different nanoslit widths and (B) the extracted reflection spectra obtained from the CytoViva hyperspectral imaging measurements.

In experimental measurement, unpolarized plain light (broadband) from a halogen lamp was used as incident light to illuminate the nanoslit samples from the top. The reflecting light signal captured by a 10× objective lens (NA = 0.3) is then collected using a darkfield CytoViva hyperspectral imaging system. Figure 3 presents the reflection spectra of the nanoslit arrays at different slit widths from both the FDTD simulation and experimental measurements. Figure 3A shows the relative reflectance of the calculated results, and Figure 3B is the extracted reflection spectra from the hyperspectral images. The relative intensity change and peak position provide corroborative information on the nanoslit array optical property and the dependence on the nanoslit width. Specifically, at nanoslit widths of 50, 70, 90, 110, and 130 nm, the primary resonant peak position is changed from 609, 615, 621, 649, to 755 nm obtained from simulation while experimental peak positions obtained at 585, 590, 620, 655, and 738 nm correspond to the nanoslit width of actual samples measured to be 55.38, 73.6, 90.1, 115.2, and 135 nm. The results indicate a good agreement between the simulation and experimental results. The slight difference between the simulated and experimental peak positions may be due to the imperfection of nanoslits during pattern fabrication and the small deviation of the actual nanoslit widths from the simulation. This is expected because the slit width determines the composition of polariton states.^30^ The simulation shows two reflectance peaks (1 and 2), which may be assigned two different plasmonic modes SPP and LSPR, respectively.^34^ The experimental reflection spectra represent the combined modes but are dominated by the primary plasmonic peak 2. One can see that the resonance peak red-shifts with respect to an increase in slit width with the decrease of the field intensity. The ability to tune the resonant wavelength position is particularly important in investigating the light–matter interaction where the absorbing material can be excited at a specific wavelength. In brief, the simulation and experimental results validate the geometry dependence for the plasmonic resonance of the nanoimprinted gold nanoslit arrays at the PDMS substrates.

To further understand the relationship of surface plasmon to the optical enhancement, we applied a previously reported semianalytical mode to calculate the surface plasmon generation efficiency (SPGE) for different nanoslit widths (see details in the SI).^56^Figure S3 shows a correlation between the SPGE and EM field obtained in FDTD and experiments from Cytoviva hyperspectral imaging. In the nanoslit array, its SPGE approximately has the same dependence as the EM field at the NS surface on the nanoslit width. The correlation of the SPGE and optical field intensities with respect to the geometric parameters provides us with a guide for the development of advanced optical devices.

### Optical Enhancements of Dye Molecules on the Gold Nanoslit Arrays at the PDMS Substrate

In this study, the optical enhancement of dye molecules at the nanoslit array substrates was examined. In contrast to previous work where the bottom of the nanoslit is a nonmetallic component,^32,33^ here, the in-slit bottom is designed to contain a thin film of Au (Figure 2). This configuration creates an extra metal-dielectric interface that provides an additional interface for SPP excitation for improved interaction with excitons and offers an avenue for plasmonic manipulation.^20^ Additionally, the in-slit Au bottom system was chosen because it supports broadband resonance reflection, which is expected to provide efficient excitation and emission of PL (Figure 3B). The emission quenching that typically occurs when emitters are in close or direct contact with metals is prevented here since the dyes are sandwiched between the high electric fields generated by the adjacent walls and the bottom of the nanoslit.^33^ There are other approaches that attempt to provide optical enhancement due to plasmon–exciton coupling in nanostructures, such as core–shell nanoparticles or nanocone cavities.^57−59^ Nevertheless, there is a need to have a better understanding of the underlying reaction mechanism of different NS designs, thus improving the optical signal enhancement. To address the need, we investigated the impact of excitation and emission wavelengths of fluorophores with excited plasmons in the nanoslit array. To determine the excitation/emission wavelength requirement necessary for optimum coupling of a fluorescence molecule within the plasmonic field, a dye–Au nanoslit hybrid system was constructed (see the Experimental Section) and the resulting optical field enhancement responses were quantified. The intensity and position of SPP resonances from the reflection spectra of the patterned samples and references containing AO, PI, and DHE were measured using a CytoViva hyperspectral imaging system. Figure S4 shows representative net intensity spectra measured for AO, PI, and DHE in the absence of nanoslit (black dotted line) and in nanoslit of different widths. The net reflection spectra were obtained by subtracting the background without the dye. It is assumed here that the plain light source at normal incidence to the nanopattern excites plasmons λ_**p**_, which then interact with the excitation and/or emission of the dye molecules. The measured reflectance thus represents a joint contribution of the processes of plasmon evanescence, light scattering, and the fluorescence emission of the dye. To investigate how the net reflectance intensity is controlled by these processes in the nanohybrid structure, we investigate the relationship of reflectance to the plasmon-absorbance match (Δλ_**1**_ = |λ_**p**_ – λ_**abs**_|) referred to as excitation enhancement and the plasmon-emission match (Δλ_**2**_ = |λ_**p**_**–** λ_**em**_|) referred to as the emission enhancement. The smaller Δλ represents the more spectral overlap of plasmon resonances with molecular absorption or emission resonances. Figure S5 shows the normalized optical reflectivity of the device together with the absorption and emission features of AO, PI, and DHE. For AO, Δλ_**1**_ increases from 103 to 243 nm and Δλ_**2**_ from 62 to 202 nm as the slit width increases from 50 to 130 nm, respectively. A similar trend is observed for DHE showing Δλ_**1**_ and Δλ_**2**_ increases from 238 to 378 nm and from 170 to 310 nm, respectively. PI displayed Δλ_**1**_ from 148 to 288 nm and a very strong plasmon-emission overlap with the Δλ_**2**_ ranging from 3 to 108 nm in the different nanoslit widths. Overall, AO shows a better excitation overlap (smallest Δλ_**1**_) in all nanoslit structures whereas PI exhibits the best plasmon-emission overlap (smallest Δλ_**2**_). It is therefore convenient to assume that any modification in the optical characteristic of AO and PI by the plasmon band occurs via plasmon-absorption or plasmon-emission interaction or both. DHE shows very wide Δλ**_1_** values and the plasmon-absorbance spectra do not overlap at all.

The impact of these overlaps on the reflectance intensity enhancement factor (INF) *I*_E_ in different slit width nanostructures was obtained by normalizing the overall measured reflectance with that of the unpatterned area as a reference using eq 1.^60^
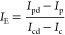
1where *I*_pd_, *I*_p_, *I*_cd_, *and I*_c_ are the measured peak intensities of nanoslit arrays with dye and without dye and an unpatterned area with dye and without dye, respectively. As summarized in Table 1, the magnitude of *I*_E_ shows dependence on the position of the plasmon band relative to molecular resonances. Typically, the *I*_E_ for AO, PI, and DHE in the different nanoslit width systems is diminished as the mismatch (Δλ_**1**_ or Δλ_**2**_) between the plasmon band and the molecular resonances of the fluorophores becomes larger. For example, AO in the 50 nm slit showed the highest *I*_E_ (56.92-fold), which decreases sharply to 4.72-fold in a *W* = 130 nm structure. The decrease in *I*_E_ could be caused by either the decrease in the electromagnetic field intensity in the different nanoslit structures (Figure 3) or a plasmon-molecular resonance mismatch. To verify which effect is at play, we quantified and compared the percentage change in intensity of the different width nanostructures relative to the 50 nm structure without and with molecular dyes. The percentage change in intensity, Δ*I* is calculated using the equation.^61^
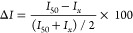
2where *I*_50_ is the maximum peak intensity in the 50 nm structure and *I*_*x*_ is the maximum peak intensity in the other nanostructures. Δ*I* shows the value in percentage by which the intensity changes as the slit width is increased from 50 to 130 nm. Relative to the 50 nm slit width structure without a molecular dye, the 70, 90, 110, and 130 nm width structures show a decrease in intensity by 13, 53, 87, and 147% respectively (Table S1).

**Table 1:** Optical Intensity Enhancement and Spectral Mismatch between Plasmon and Molecular Resonances in Different Width Structures Containing AO, DHE, and PI

slit width	Abs (Δλ**_1_**) or Em (Δλ**_2_**)-plasmon mismatch (nm)						intensity enhancement factor (INF)		
	(AO)		(PI)		(DHE)		*I*_E_		
*W* (nm)	Δλ**_1_**	Δλ**_2_**	Δλ**_1_**	Δλ**_2_**	Δλ**_1_**	Δλ**_2_**	AO	PI	DHE
**50**	103	62	148	32	238	170	56.92	24.42	1.94
**70**	103	62	148	32	238	170	48.25	24.12	1.32
**90**	132	91	177	3	267	199	15.43	30.01	0.06
**110**	167	126	212	32	302	234	7.97	22.28	0.02
**130**	243	202	288	108	378	310	4.72	2.29	0.01

With AO coupled to the nanostructures, the intensity decreased by 16, 102, 137, and 165%, respectively. By comparing the percentage change in intensity without and with AO dye for each NS, we see a positive increase in the presence of AO. For example, the Δ*I* in a 70 nm structure without the dye, minus the Δ*I* in a 70 nm structure with the dye is 2%. This kind of difference, which is observed in all of the nanostructures, is ascribed to the spectral mismatch effect. Therefore, the corresponding increase in the intensity enhancement factor for AO (Table S1b) as the slit width decreases shows the dependence of *I*_E_ on the interplay between the excitation/excited state of the dye and the SPR.^62^

The low optical enhancement in the DHE system relative to AO and PI (see Table 1) is due to a large mismatch of the plasmon resonant peaks from DHE’s absorption and emission wavelengths. Since the spectral overlap of the fluorophore with plasmon resonance is responsible for the coupled system emitting a photon, a highly mismatched system probably leads to the reabsorption of energy and dissipating it as heat.^63^

For PI in the 90 nm slit width, the optical enhancement is 30-fold, which is more than the enhancement in all PI-hybrid systems including 50 and 70 nm width nanostructures. The observation can be traced back to the strong plasmon-molecular emission spectral overlap (Δλ_**2**_ = 3 nm) in W = 90 nm, which compensated for the relatively lower field in the 90 nm slit width compared to 50 and 70 nm slit width structures. It can be inferred that the *I*_E_ in the PI-hybrid system is mainly controlled by plasmon resonance with emission. The higher *I*_E_ (56.92-fold) in the AO system relative to the PI system (24.42-fold) may be attributed to the close absorption and emission spectral overlap between plasmon and molecular resonances. In brief, these findings demonstrate that the plasmon–exciton spectral overlap is highly critical for optical signal enhancement in this type of plasmon–exciton hybrid system.^20,30,64^ The observed behavior indicates an interplay of both excitation and emission enhancements induced by the plasmon mode in Au nanoslit.

### DNA Assay

Acridine orange was chosen for this assay because of its above-observed superior optical enhancement in the nanoslits. The cationic property of amino-modified acridine offers a suitable basis for DNA probe assembly. Negatively charged ssDNA was introduced to 9-aminoacridine which was cross-linked via EDC/NHS to the gold nanoslit array at a PDMS substrate to generate signals corresponding to targets. The assembly was induced by electrostatic interactions between ssDNA and AO (see method section). In order to obtain signals that will discriminate between ssDNA and dsDNA, it is necessary to select a plasmon resonance whose wavelength position overlaps well with the emission of AO-ssDNA or AO-dsDNA (Figure S5). We chose emission overlap because of its overwhelming effect at increasing the rate of radiative recombination of the exciton state. Excitation overlap on the other hand could increase the population of excited states but a portion of it could undergo nonradiative loss to generate heat that might affect the biological analyte. Hence, we selected a 110 nm structure whose resonance wavelength position at peak λ = 655 nm is close to the emission resonance of the AO-ssDNA complex (λ = 650) and more mismatched with the AO-dsDNA complex.

Figure S6A shows that the introduction of ssDNA to acridine in the NS resulted in the further enhancement of the optical field previously recorded for acridine alone in the nanoslit structure (black vs. blue curves). In the presence of a complementary target DNA (ct-DNA), the intensity drops back down (red curve) due to the hybridized dsDNA. This behavior offers a discriminatory approach to the detection of ssDNA and a hybridized dsDNA.

To ensure that the optical signal changes are not due to the direct interaction of the free or unreacted DNA probe or target with plasmons in the nanoslit or via nonspecific binding, we carried out a separate experiment where ssDNA (without AO) is incubated with the Au nanoslit that has been previously modified with SAM of 11-mercaptoundecanoic acid and 8-mercaptooctanol. As expected, Figure S6B shows that the plasmon field intensity does not show any significant change proving that there is no interaction between DNA and the nanoslit surface, confirming the important interplay role of the AO dye with the DNA and nanoslit plasmon for optical signal differentiation.

**Figure 4:**
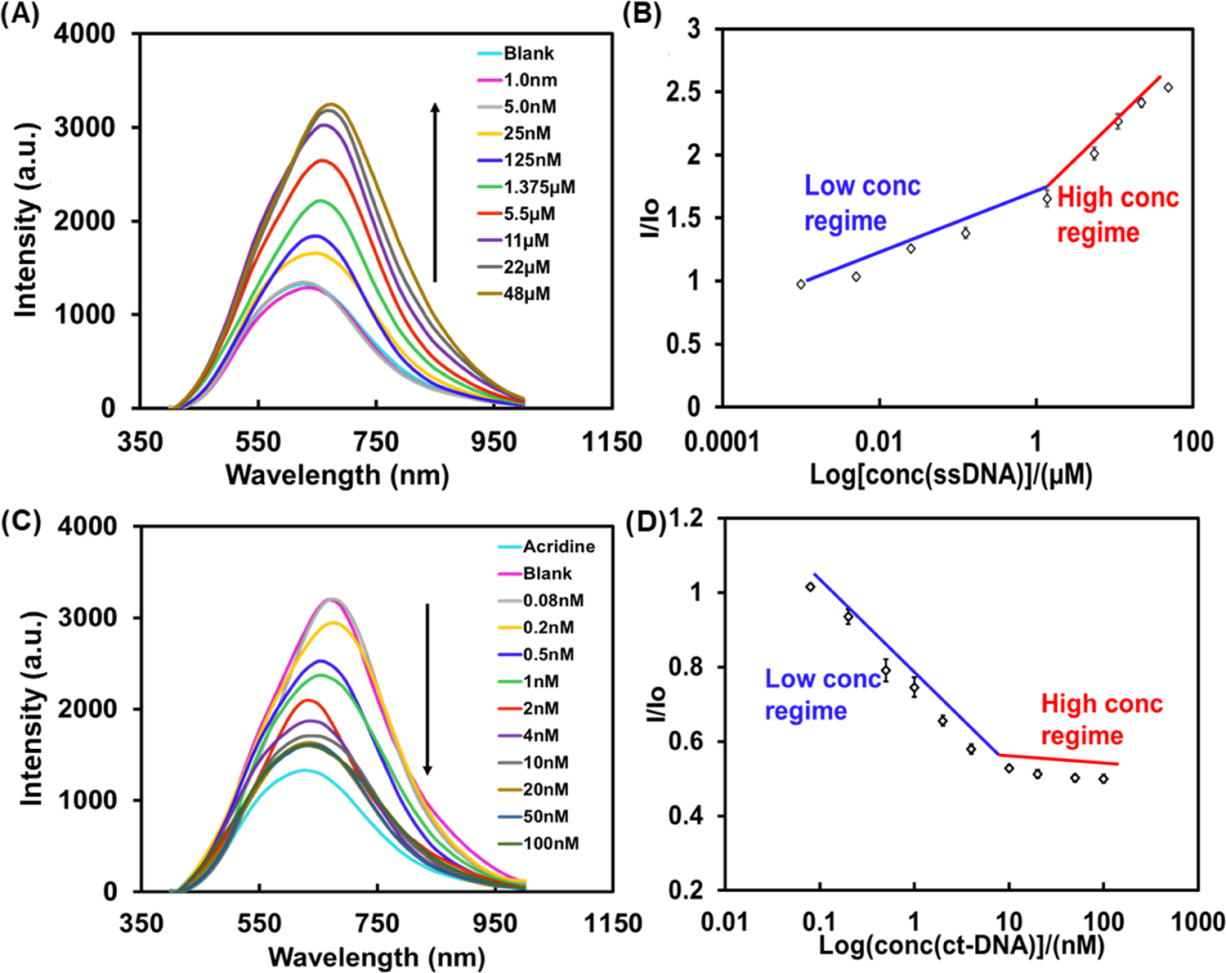
Interaction of DNAs with AO in the 100 nm nanoslit arrays. (A) Field intensity spectra with different concentrations of ssDNA. (B) Plot of *I*/*I*_o_ versus the log concentrations of ssDNA. Note that *I* and *I*_o_ are intensities of AO in the NS before or after adding ssDNA. (C) Field intensity spectra of AO-ssDNA with different concentrations of complementary target DNA (ct-DNA). (D) Plot of *I*/*I*_o_ versus the logarithm concentrations of ct-DNA.

Acridine orange is not specific or selective to the ssDNA. This means that the capture ssDNA probe can bind to AO, and the target DNA can also bind to AO to cause an unspecific binding signal, respectively. To prevent this, it is necessary to determine the minimum equilibrium ssDNA concentration where the AO active surface is no longer receptive to binding DNAs. Figure 4 shows the spectral peak variation and calibration curves obtained for the interaction of AO with different concentrations of the capture ssDNA and the target ct-DNA molecule. Figure 4A exhibits a gradual increase in the intensity of AO-plasmon spectra as the concentration of ssDNA is increased from 1 nM to 22 μM. Little intensity fluctuation is noticed above 22 μM, suggesting saturation of the ssDNA binding at the AO surfaces. For this reason, a concentration of 22 μM or larger ssDNA probes was used for the preparation of sensor substrates in subsequent experiments. The consequent effect of using the below-equilibrium point concentration (less than 22 μM) of the ssDNA probe on AO is shown in Figure S7. The addition of 4 nM ct-DNA to the AO-ssDNA substrate made from the 4 μM ssDNA capture molecule resulted in optical signal intensity enhancement due to the direct interaction of ct-DNA with only partially ssDNA-saturated AO.

Further, it was found that the optical signal depends on the concentration of ssDNA in two different regimes. Figure 4B shows linear relationships between optical changes and the concentration of ssDNA. At a high-concentration regime, AO and ssDNA interact much more rapidly via dye-base stacking to form the AO-ssDNA complex.^50^

Next, considering the specific interactions between ssDNA and its complementary counterpart, we tested the optical signal transduction mechanism due to the optical signal differences of the AO-ssDNA interaction and DNA hybridization. We took advantage of the phenomenon of field enhancement and introduced the ct-DNA to the AO-ssDNA complex. Figure 4C shows the optical signal quenching with the addition of ct-DNA up to 100 nM until no further diminish in the signal intensity is observed. The calibration curve for ct-DNA detection was determined based on serial concentrations of the ct-DNA at 0.08, 0.1, 0.2, 0.3, 0.5, 1.0, 2.0, 4.0, 10.0, 20.0, 50, and 100 nM. Figure 4D plots the relative signal intensity increase (*I*/*I*_o_) versus the logarithm value of the ct-DNA concentration that displays two linear ranges consisting of 0.08–10 nM (*R*^2^ = 0.994) and 20–100 nM, respectively. The limit of detection (LOD) was evaluated based on the relation, LOD = 3σ/*S*, where σ is the standard deviation of the blank signal and *S* is the slope of the calibration plot.^21^ The LOD was found to be 0.21 nM at a signal-to-noise ratio of 3 in the linear range of the low-concentration regime. The low-concentration range relatively shows a rapid decline in intensity, which slows down and reaches a steady state at higher concentrations. This is because, at low concentrations, free monomers ssDNA start hybridizing with ct-DNAs to form dimers nearly instantaneously with a faster reaction rate.^65^ As free monomers become depleted due to hybridization with ct-DNA, further increases in the ct-DNA concentration produce a smaller intensity response until there is no detectable signal change. At the concentration (100 nM), the ssDNA is in equilibrium with the ct-DNA. Thus, the difference in linearity between the low-concentration regime (0.08–10 nM) and the high-concentration regime (20–100 nM) arises from a decrease in binding kinetics that occurs at a concentration above 10 nM.^66,67^ The linear range and the low LOD shown here demonstrate that our proposed approach may meet the sensitivity requirement for the DNA hybridization assay. A detailed comparison of this work with other SARS-CoV-2 biosensors using different methods is presented in Table S2. The LOD and detection time of this work is superior or competitive to recently reported results.

**Figure 5:**
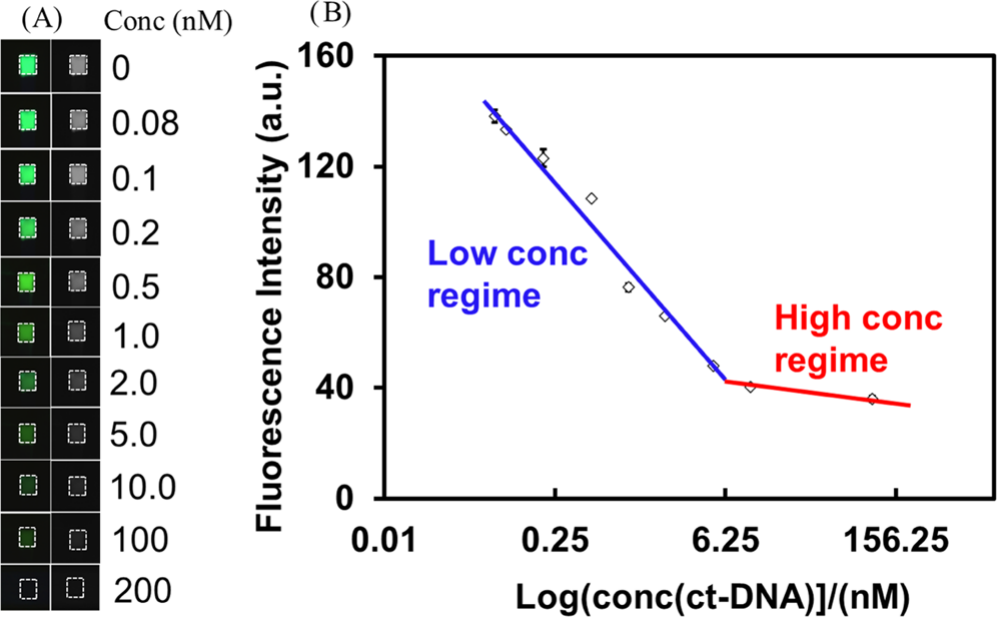
Fluorescence images at different concentrations of ct-DNA in nanoslit arrays. (A) Images taken using a 475 nm excitation light source and a 546 nm cut-off filter. Note that on the right side are the images when converted to grayscale. (B) Graphic plot showing fluorescence intensity as a function of ct-DNA concentration ranging from 0.08 to 200 nM.

In addition to the hyperspectral signal study, we further examined fluorescence changes using an Axio Z2M imaging microscope at 475 nm excitation. Figure 5A shows the fluorescence images of AO-ssDNA probe substrates in the presence of ct-DNA at different concentrations. It can be visualized that at higher concentrations of ct-DNA, the fluorescence intensity decreased. Note that the emission from the AO-ssDNA probe (at a peak around 650 nm) is blocked by a 546 nm cut-off filter. From the grayscale images in Figure 5A, the fluorescence intensities corresponding to the concentrations of the ct-DNA were obtained. Figure 5B displays a linear dependence of the fluorescence intensity on the logarithm value of the ct-DNA concentration in a range of 0.08–200 nM, a good agreement with the net reflectance intensity as a function of the ct-DNA concentration (Figure 4D). The error bars convey the average fluorescence signal over a 25 min period after the reaction equilibrium is reached.

**Figure 6:**
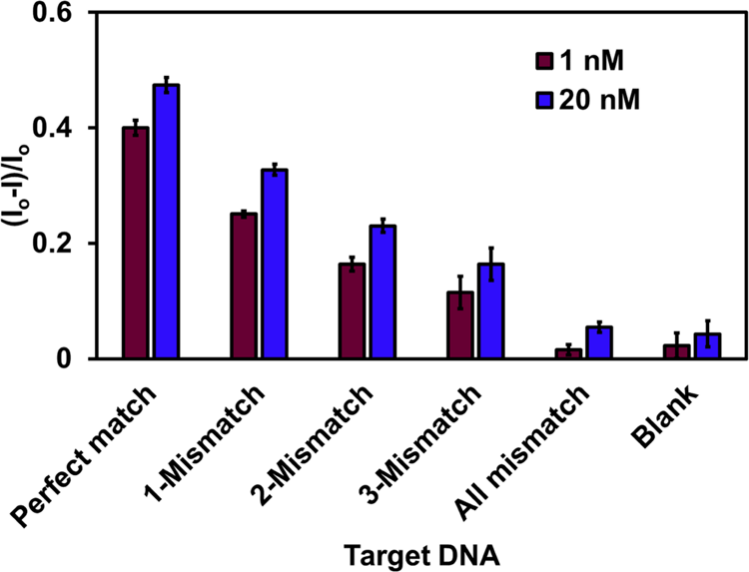
Normalized fluorescence upon addition of 1 and 20 nM perfectly matched DNA with different mismatched bases.

Using noncomplementary target DNAs as control experiments, the specificity of the assay was investigated using 1-, 2-, 3-mismatched, and all mismatched DNA samples, respectively. Figure 6 shows the relative fluorescence intensity changes of perfectly matched ct-DNA as a comparison to the mismatches for both 1 and 20 nM concentrations. The results indicate a satisfactory selectivity by providing a significant signal change to differentiate even one base mismatch. Figure S8 shows the fluorescence images of AO and AO-ssDNA in the absence and presence of ct-DNA on the PDMS substrate without nanostructures and their red, green, and blue (R, G, B) intensity profile. The color of the fluorescence images changes from green for AO to red (for AO-ssDNA) and finally back to green after hybridization with ct-DNA. This means that the decline in the resonance intensity peak due to the ct-DNA hybridization with the probe DNAs occurs because the emissive capability of the acridine-dsDNA complex in the nanoslit structure is shifted significantly to the green region, which is away from the plasmon resonance. This tends to weaken the coupling effect of the dye with resonating plasmons generated in the nanoslit arrays.

### Stability and Reusability of the Sensor

In addition to sensitivity requirements, high stability is crucial to enable easy storage and deployment of the biosensor without necessarily compromising its integrity for precise sample detection. To evaluate the stability, the sensor device was kept at room temperature for up to 10 days and the optical response of the sensing system was monitored before and after hybridization with 0.5 nM ct-DNA. Figure S9A indicates that the hybridization signal intensity on the first day is consistent with the one observed after 10 days. In addition, we performed the regeneration of the sensor surface after hybridization, Figure S9B demonstrates the reusability of the sensor after hybridization. Notably, the active probe surface sustains two successive assays after repeating the regeneration without any significant loss of performance as indicated by the recovered signal intensity of the probe. After the second regeneration cycle, there is a noticeable increase in the hybridization signal intensity, which suggests an incomplete DNA interaction. This occurrence may be due to the weakening of the DNA binding affinity, resulting from the multiple heating and cooling, which presumably affects the stability of the probe monolayer, and thus a slight change of the density of AO-ssDNA. However, the regeneration of the probe allows for the sensor to be reused 6 times with a mean recovery of 96.54% of the original signal. This proves that the fabricated nanoslit array sensor is not only stable but reusable without necessarily repeating the surface functionalization step.

Herein, the principle of this work for the detection of DNA hybridization at the flexible plasmonic nanoslit array substrates was validated and demonstrated. The metachromatic property of acridine dye was employed for the quantification of nucleic acid hybridization in a plasmonic environment. As shown in Figure 1, the overall strategy and mechanism lead to the discrimination of ssDNA and dsDNA at the nanoslit sensing area in optical responses. Initially, ssDNA is saturated onto the cationic acridine-modified surfaces via electrostatic interactions causing the emission wavelength of acridine to shift from 525 to 650 nm, which matches the plasmon resonance. Such a plasmon–exciton coupling effect causes significant optical field enhancement and signal amplification. The addition of ct-DNA to this substrate diminishes the optical signal in the nanoslit structure in the measured reflective hyperspectral and fluorescence images. This occurs because the hybridization of the capture DNA probes with the ct-DNAs forms a dsDNA and causes the emission wavelength shift of the AO-DNA complex from 650 to 525 nm, which is away from plasmonic resonance energy, thereby reducing the plasmon–exciton coupling effect. The increase of optical signal in the presence of ssDNA and the signal decrease in the presence of ct-DNA upon hybridization provides the highly responsive sensing capability of the device. This work demonstrates a significant improvement in signal amplification using a well-designed plasmon–exciton coupling system for the development of a highly sensitive device for detecting DNA hybridization and binding studies.

## Conclusions

In summary, we have used both computational modeling/simulation and a simple nanoimprinting technique for developing plasmonic nanoslit arrays at a flexible PDMS substrate and demonstrated a plasmon–exciton coupling effect on a surface-modified dye for optical enhancement and DNA hybridization detection and its capability to differentiate single-base mismatch. The reported nanoimprinting fabrication process for hybrid nanostructured arrays based on gold-coated PDMS renders the generation of various geometries and nanoslit sizes by controlling the thickness of the Au layer without the need for expensive or sophisticated patterning instruments. The plasmonic resonance peak wavelength of the nanoslit arrays was tunable by varying the nanoslit width from 50 to 130 nm. The coupling of the plasmonic field with a molecular exciton was found to boost the PL of dye molecules attached to the nanoslit surfaces when the excitation and emission energy of the molecule matches well with the plasmon resonance energy. The 50 nm plasmon slit array with an AO exciton hybrid system showed a maximum optical field enhancement of 57-fold and was used to monitor DNA–DNA hybridization based on the different emission wavelengths of the AO-ssDNA and AO-dsDNA systems upon hybridization. The device was evaluated using 1-, 2-, or 3- base mismatches in the detection of DNA hybridization, which showed significant signal differentiation for high sensitivity and specificity and the LOD of 0.21 nM ct-DNA for perfectly matched DNA hybridization. It also demonstrated high stability and can be reused six times with about 96% signal recovery. This contribution that provides a model of sensing applications using metachromatic fluorescence dyes of plasmonic signal amplification for DNA detection will further stimulate broader applications of the sensing method in clinical determination and biological analysis. It was found that the in-nanoslit Au film played a key role in localized field enhancement and thus the amplification of the optical signal for sensing application. Owing to the simple fabrication and functionalization approach, such a plasmon–exciton coupling method will play a significant role in developing next-generation optical sensors.

## Experimental Section

### Chemicals and Materials

Dihydroethidium (90% purity, Cat. no. D11347), propidium iodide (94% purity, Cat. no. BMS500PI), phosphate-buffered saline buffer (PBS, 10 mM PB, 150 mM NaCl, pH 7.4, Cat. no. 003002), poly(dimethylsiloxane) prepolymer (Dow Corning Sylgard 184), and absolute ethanol (Cat. no. T038181000) were purchased from Thermo Fisher Scientific. Acridine orange dye (Cat no. 10127-02-03), 11-mercaptoundecanoic acid (11-MUA, 95% purity, Cat. no. 450561), 8-mercapto-octanol (8-MA, 95% purity, Cat. no. 075075), 1-ethyle-3-(3-dimethylaminopropyl) carbodiimide hydrochloride (EDC, 99% purity, Cat. no. 03449), *N*-hydroxysuccinimide (NHS, 98% purity, Cat. no. 130672), and perfluorinated trichlorosilane (97% purity, Cat. no. 448931) were purchased from Sigma-Aldrich. Silicon mold from II–VI Aerospace & Defense, gold (99.99% purity, Cat. no. EVMAUXX), and titanium (99.99% purity, Cat. no. EVMTI45EXE-J) were obtained from Kurt J. Lesker. Table 2 shows the HPLC-purified oligonucleotide probe and targets that were obtained from Integrated DNA technologies. All chemicals and materials were used as supplied without any modifications.

**Table 2:** DNA Probe and Targets^a^

name	sequence (5′-3′)
oligo 1 (probe)	ACA CAC GCA TGA CGA CGT TAT AACA
complementary target	T GTT ATA ACG TCG TCA TGC GTG TGT
1-mismatched target	T GTT **T**TA ACG TCG TCA TGC GTG TGT
2-mismatched target	T GTT **T**TA ACG TCG TCA TGC **C**TG TGT
3-mismatched target	T GTT **T**TA ACG **G**CG TCA TGC **C**TG TGT
noncomplementary target	G TGC TAG CTA ATC AGC ACT AAA GTA

a The DNA probe was designed to target a genome region (2630–26321) of severe acute respiratory syndrome coronavirus 2 isolates (SARS-CoV-2/human/USA/LA–BIE-092/2020) whose sequence is used here as the complementary target.

### Instrument and Equipment

The excitation and emission/fluorescence spectra of numerous solutions were obtained on a Varian Cary eclipse fluorescence spectrophotometer (Agilent). SEM images were taken on a field-emission scanning electron microscope (Carl Zeiss Auriga). Plasmonic field reflection spectral measurements were done on a Cytoviva hyperspectral microscope. Fluorescence images were captured with an Axio Z2M (Zeiss) microscope equipped with excitation filters of 395–410 and 490–505 nm using a 20× objective lens at 5.0 s exposure time. Other instruments used include a plasma cleaner (South Bay Technologies PC2000), an e-beam evaporation tool (Kurt Lesker PVD75 e-beam evaporator), UV/ozone Pro cleaner, and an LSE–WS Stokes WAFERSKAN ellipsometer.

### Fabrication Procedure

The fabrication process is schematically depicted in Scheme S1. Briefly, a molding soft lithography is performed on a Si template consisting of a nanoslit array to produce a negative pattern of the Si template on PDMS. This is achieved by curing and careful peeling of PDMS off the Si template. Finally, a deposit of a thin metal film of desired thickness was made on the top surface of the PDMS mold by electronic beam evaporation.

### Pattern Transfer of a Periodic Si Nanoslit Array

A patterned Si wafer was used as a master mold used to transfer a periodic nanoslit array onto a flexible PDMS (Figure S1). The Si wafer has a nanoslit array of periodicity and slit depth of 300 and 150 nm, respectively. The surface of the silicon wafer was salinized to ensure easy peeling of cured PDMS. Briefly, 1 mL of perfluorinated trichlorosilane in an aluminum dish was put into a desiccator alongside the patterned wafer held by scotch tape in a Petri dish. The desiccator was pumped down for 6 min and the pump was turned off. The wafer was allowed to stand in the desiccator for 6 hours. Meanwhile, the PDMS for pattern transfer was obtained by mixing a 10:1 weight ratio of the polymer base and cross-linking agent (curing) at room temperature. The mixture was degassed in a desiccator using a rotary pump. The bubble-free PDMS mixture was then poured onto the patterned Si wafer and baked in an oven for 1 h at 75°C followed by cooling. Finally, the dried sample was peeled to obtain a patterned PDMS substrate.

### Gold Deposition on the Patterned PDMS Substrate

The PDMS was first treated with oxygen plasma (power density, 20 mW/cm^2^; pressure, 100 mTorr; O_2_ flow, 10 sccm; Ar flow 40 sccm; exposure time 5 s) to enhance metallic adhesion onto the surface. The PDMS template, physically supported on the back of a watch glass (60 mm diameter, 2 mm thick, 6 mm depth) was coated first with 5 nm titanium, then with gold via e-beam evaporation at a rate of 1.6 Å/s to the desired thickness at a vacuum pressure of 8.2 × 10^–6^ Torr. The thickness was monitored and measured using a quartz crystal microbalance (QCM). To improve the uniformity of the deposited Au material, the sample plate was rotated throughout the deposition process. The thickness of the deposited gold was further verified using an ellipsometer.

### Preparation of the Dye-Modified Au Nanoslit

Three different dyes (acridine orange, propidium iodide, dihydroethidium) having different optical properties were each prepared to a concentration of 0.5 mM. Next, the nanoslit-cavity devices were cleaned with 15 min exposure to UV/ozone. Then, 50 μL of each dye was drop-coated onto a gold nanoslit array. The dye was allowed to dry in a dark environment and was immediately used for optical studies. The fluorescence images before and after dye coating (Figure S2) expressed dye modification of the Au surface.

### Preparation of Immobilized AO onto the Gold Nanoslit Array

First, gold-coated PDMS chips with nanoslit arrays were cleaned with O_2_ plasma for 10 min. Next, a self-assembled monolayer (SAM) was created on the surface of the PDMS arrayed chips by incubating it in a 1:2 mole ratio of 11-MUA, and 8-MA, in an absolute ethanol solution for 24 h. The carboxylic functional groups of the SAM were then activated by incubating the chips in a 0.5 mM EDC/NHS for 2 h. The chips were then rinsed with DI water and immediately submerged in 5 mL solution of freshly prepared 20 μM amino-modified AO in aqueous PBS for 2 h. The chips were rinsed with DI water, dried, and kept in a dark cupboard before the next experiment.

### Preparation of the AO-ssDNA Probe in a Gold Nanoslit Array

The ssDNA solution was prepared by resuspending the lyophilized powder of DNA oligo (101.7 nmol) in 1.017 mL of 0.1 mM PBS (pH 7.4) to obtain a 100 μM stock. From the stock, further dilutions were made to obtain the desired concentration. A 100 μL aliquot of ssDNA of desired concentrations (1.0 nM, 5 nM, 25 nM, 125 nM, 1.375 μM, 5.5 μM, 11 μM, 22μM, 48 μM) was transferred onto the chip sensing area and incubated at room temperature for 2 h in a dark environment. The chip was rinsed with DI water, dried using nitrogen gas, and stored in a dark environment for further experiments.

### Detection of Target DNA in Gold Nanoslits

The dried and HPLC-purified target DNA (90.2 nmol) was first resuspended in 902 μL of PBS (pH 7.4) to make a 100 μM stock solution. Further dilution was made to obtain the desired concentrations. Under optimal conditions of the amino-modified AO-ssDNA probe, desired concentrations (0.08, 0.2, 0.5, 1.0, 2, 4, 10, 20, 50, 100 nM) of 100 μL of the target DNA were incubated with the probe complex for 25 min. Next, the chip was rinsed with DI water before taking measurements. Note that as a check for the assay’s specificity, we used mismatched DNA targets. The noncomplementary target DNA was used as a control to further demonstrate the selectivity of the sensing method for a perfectly matched DNA target. This method shows a distinguished signal for the single-base mismatch at room temperature.

### Regeneration and Reusability of Probe Films

To regenerate the active sensor surface (AO-ssDNA complex) after hybridization, denaturation of the surface dsDNA was achieved by placing the sensor in a boiling water bath for 5 min. This was followed by rapid cooling in an ice bath for 1 minute and then allowed to reach room temperature before rinsing with DI water.^68^ The regenerated biosensor is then incubated with 0.5 nM ct-DNA for 25 min. The probe regeneration and reusability tests were performed six times on the same DNA film.

### Semianalytical Modeling

The signature of SPP-mode excitations was quantitatively recognized based on the completeness theorem, which offers useful information on the efficiency of SPP generation.^69^ For light propagating in a nontranslational system, the transverse field pattern can be treated as a linear combination of forward- and backward-traveling bounded and radiative modes.^56^ Therefore, the SPP generation efficiency *e* of the transverse electromagnetic fields for the slit geometry and for *w*/2 < *x* and *x* < −*w*/2 was estimated from eqs 3 and 4. Note that *e*_1_ = *e*_2_ since in both cases one encounters Au/air interfaces and the overall efficiency can be taken to be *e*_1_ + *e*_2_. Here, we take account of the normalized SPP excitation strength (|α^+^(*x*)|)^2^ and (|α^+^(*x*)|)^2^ for −*w*/2 < *x* < *w*/2 since there is an air–metal interface. Note that the field inside the slit consists of the downward- and upward-reflected fundamental modes since we are dealing with light diffraction by slit arrays.^69^
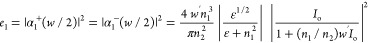
3
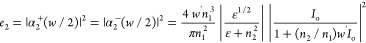
4where *n*_1_, *n*_2_, *n*_3_, and *n*_4_ are the refractive indices of the top Au–air, in-slit Au–air, and bottom Au–PDMS interfaces, respectively. The normalized slit width *w*’ = *nw*/λ where *w* is the slit width. The integrals *I*_1_ and *I*_o_ were calculated numerically for different *w*′ (Table S3) and ε for Au at different wavelengths (Table S4 and Figure S10).

### Numerical Calculations by the Finite-Difference Time-Domain (FDTD) Simulation Method

FDTD provides a numerical solution to Maxwell’s equation, which has proved useful for simulating the distribution of the electromagnetic field in complex geometries. We used a commercial software package (Lumerical Solutions FDTD) to simulate the optical transmission/reflection and electric field strength of a nanoslit array device on a PDMS substrate. The simulation domain size is 400 nm × 400 nm × 1000 nm in the Cartesian coordinates *x*, *y*, and *z*, and the time step is 5 fs. The gold film was illuminated under the plane wave transverse magnetic (TM) pulse of wavelength 400–1200 nm at normal incidence to the *x*, *y* planes (i.e., in the positive *z* direction) with an amplitude *A* = 1. To accelerate the simulation, the antisymmetric/symmetric boundary condition was used in the *x* and *y* directions, and the PML boundary condition was placed 1000 nm away from the structure in the *z* direction to absorb the back-reflected and transmitted radiation at the top and the bottom of the mesh.^70^ The mesh grid size was set to 0.3 nm in the region near the nanoslit. Gold (Johnson and Christy), with relative permittivity ε = −41.849 at a vacuum wavelength λ_o_ = 1 μm was employed.^71^ The optical field distribution and the profile of the optical field in the nanoslits were recorded by a frequency domain monitor and a 3D frequency domain profile monitor, respectively. The reflectance spectra were measured by a frequency domain power monitor placed 300 nm above the slit structure.

## Supporting Information Available

Fabrication; more SEM images; FDTD simulation results; modeling analysis; more spectra of the dye at NS surfaces; more fluorescence images; signal analysis; a comparison to other methods; and a semianalytical model for surface plasmon resonance generation (PDF)
