# Supplementary material for: Plasmon–Exciton Coupling Effect in Nanostructured Arrays for Optical Signal Amplification and SARS-CoV-2 DNA Sensing
Source: ACS Appl Nano Mater. 2023 Jan 24;6(3):2071–82. doi: 10.1021/acsanm.2c05063 (PMC9888407; doi:10.1021/acsanm.2c05063)
Supplement: Supplementary file 1 — an2c05063_si_001.pdf [file an2c05063_si_001.pdf]

Supporting Information

Plasmon-Exciton Coupling Effect in Nanostructured Arrays for Optical Signal Amplification and SARS-CoV-2 DNA Sensing

Frank Tukur, Bhawna Bagra, Anitha Jayapalan, Mengxin Liu, Panesun Tukur, Jianjun Wei

Department of Nanoscience, Joint school of Nanoscience and Nanoengineering, University of North Carolina at Greensboro

*Corresponding Author

j_wei@uncg.edu

SUPPLEMENTARY FIGURES, TABLES AND SCHEMES

Scheme S1. Fabrication steps and optimization of the Au coated nanoslit structure on PDMS via soft lithographic pattern transfer.

Fig. S1. SEM images of fabricated nanoslit array of different width size.

Fig S2. Representative EM-field distribution images from FDTD simulations.

Fig. S3. The correlation between SPGE, simulated and measured EM-field as a function of slit width.

Fig. S4. Net reflectance intensity spectra of AO. PI and, DHE in different nanoslit structures.

Fig. S5. Normalized spectral overlap between the excitation and emission spectrum of AO, PI and DHE with reflection spectrum of nanoslits of different widths.

Fig.S6. Cytoviva spectra of AO, AO and ssDNA probe in the presence and absence of ct-DNA.

Fig S7. Effect of below-equilibrium concentration of acridine-ssDNA complex on intensity signal response.

Fig S8. Axio Z2M images and corresponding RGB intensity profile of AO, AO-ssDNA and AO-dsDNA.

Fig. S9. The stability and reusability of the sensing system.

Fig. S10. Surface plasmon polariton generation efficiencies as a function of the slit width for different wavelength.

Table S1a. Percentage decrease/increase in intensity and intensity enhancement factor calculated for different nanoslit widths with respect to the 50 nm slit width**.**

Table S1b. Measured scattering intensities on nanostructure area and plain Au surface with and without dye and the corresponding intensity enhancement factor $I_{E.}$

Table S2. Sensitivity of existing biosensors for SARS-CoV-2 testing.

Table S3. Numerical data of I_0 and I_1 for different values of w' for gold at different wavelengths.

Table S4. Surface plasmon generation efficiency calculation under different incident wavelengths for different nanoslit.

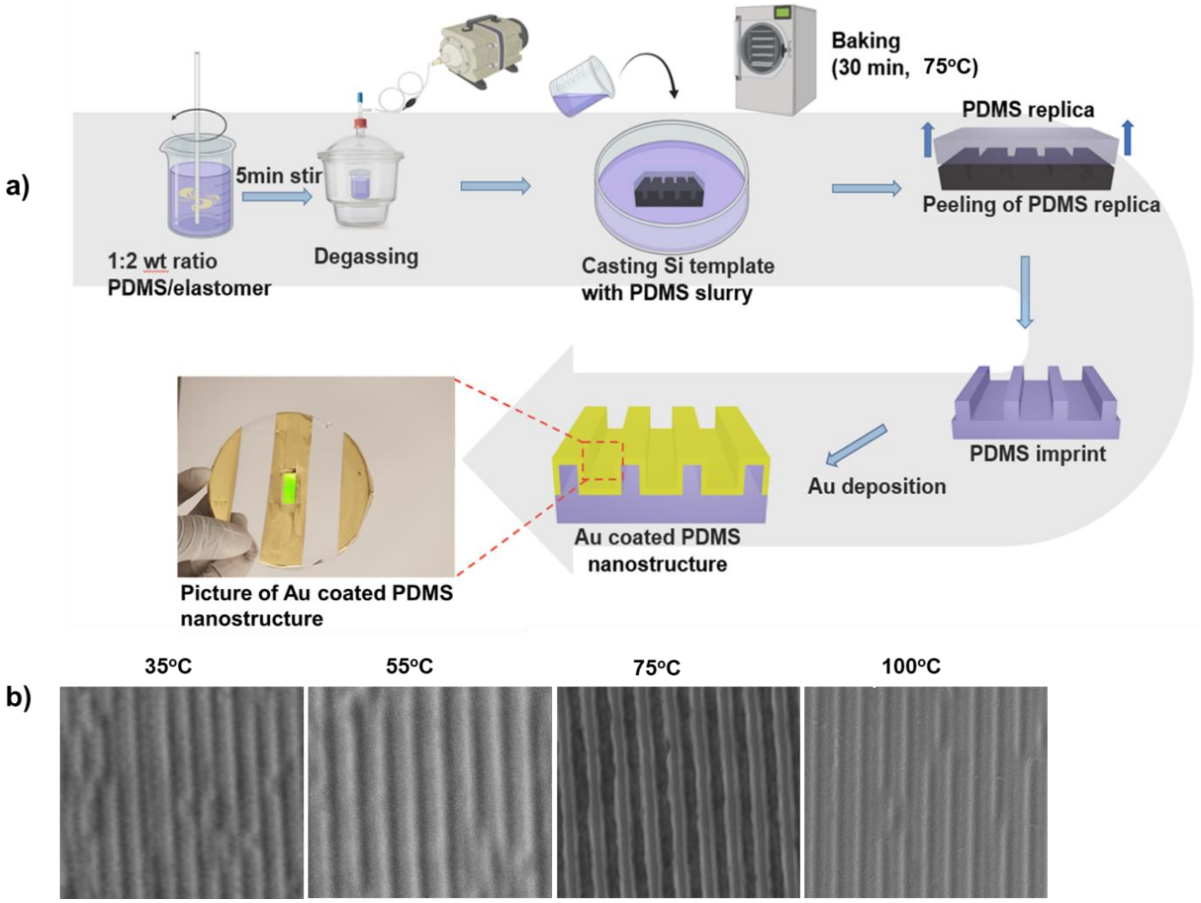

Scheme. S1. a) Fabrication steps: Au coated nanoslit structure on PDMS via soft lithographic pattern transfer. b) show the SEM images for the optimization of the nanoslit array design at PDMS baking temperatures ranging between 35ºC and 100ºC for 1 hour.

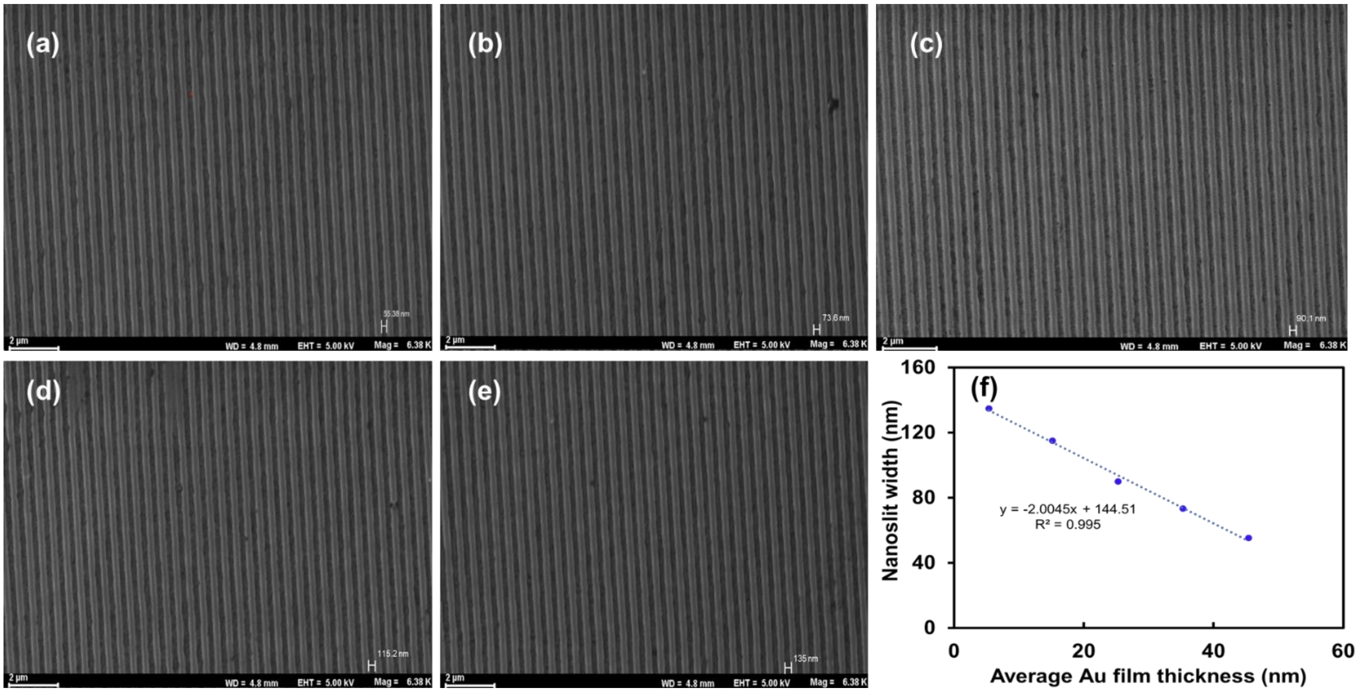

Fig. S1: SEM images of fabricated nanoslit array of width (a) 55.38 nm (b) 73.6 nm (c) 90.1 nm (d) 115.2 nm (e) 135 nm (f) show the linear relation between the measured gold film thickness (45.38 nm, 35.27 nm, 25.25 nm, 15.11 nm, 5.36 nm) with the nanoslit width.

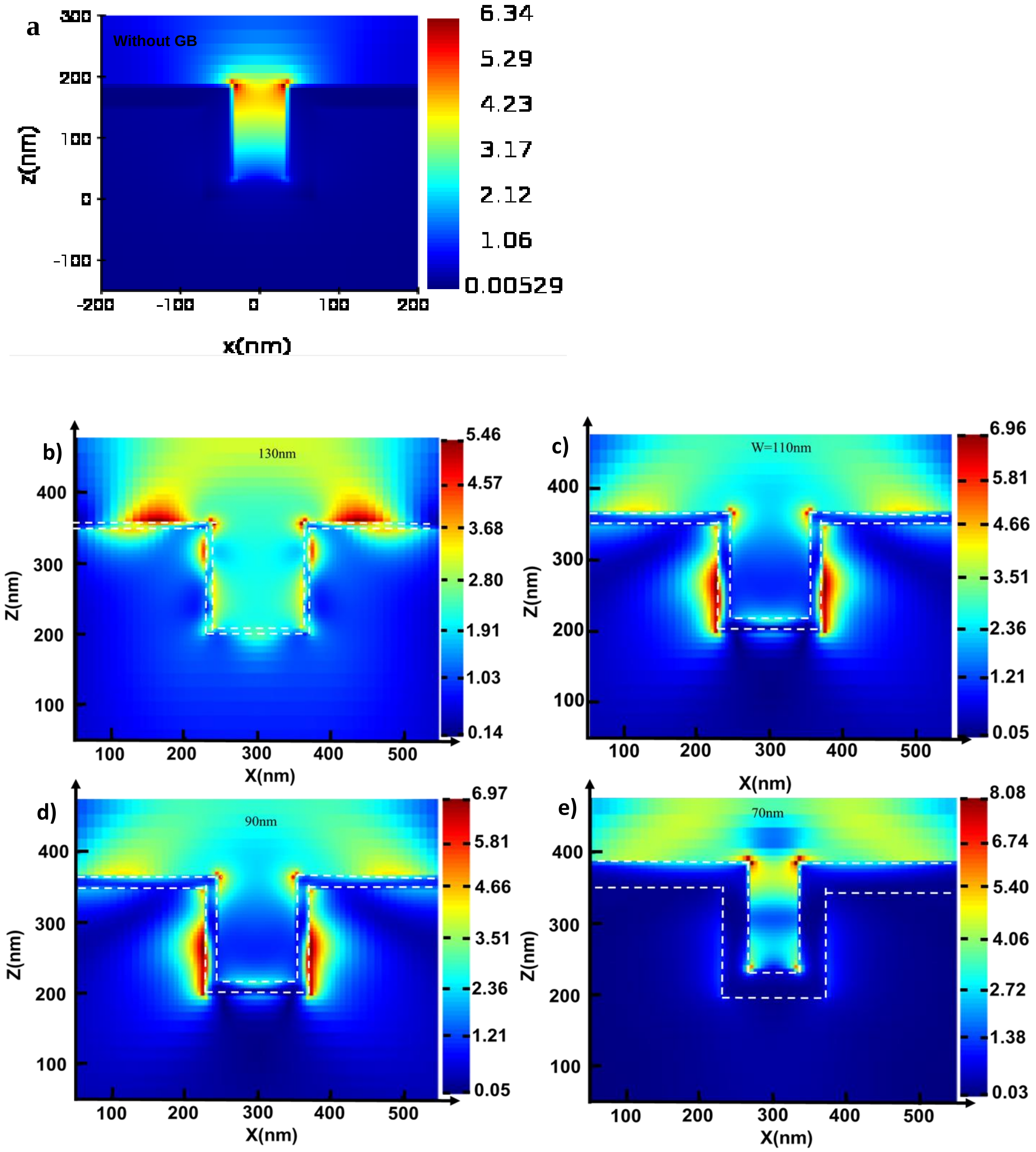

Fig. S2. Representative EM-field distribution images from FDTD simulations. We simulated a design without and with an Au film at the slit bottom. (a) shows the electric field, $|E|^2$ (V/m) ^{2} distribution in nanoslit without a gold base and (b-e) nanoslits with gold film base of different thicknesses.

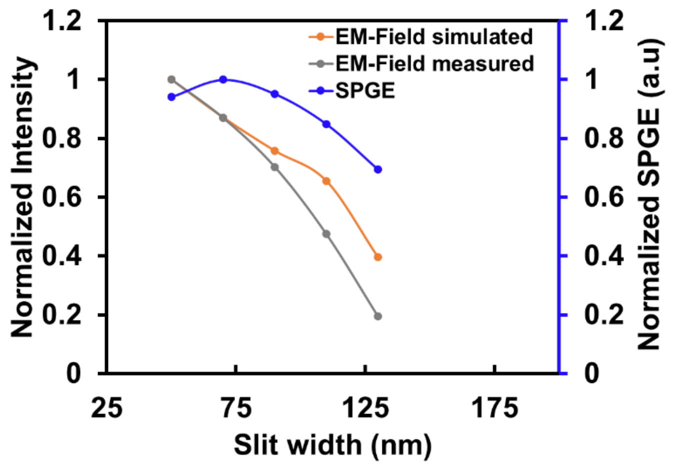

Fig. S3. The correlation between SPGE, simulated and measured EM-field as a function of slit width. Detailed description is provided in the section: semi-analytical modeling.

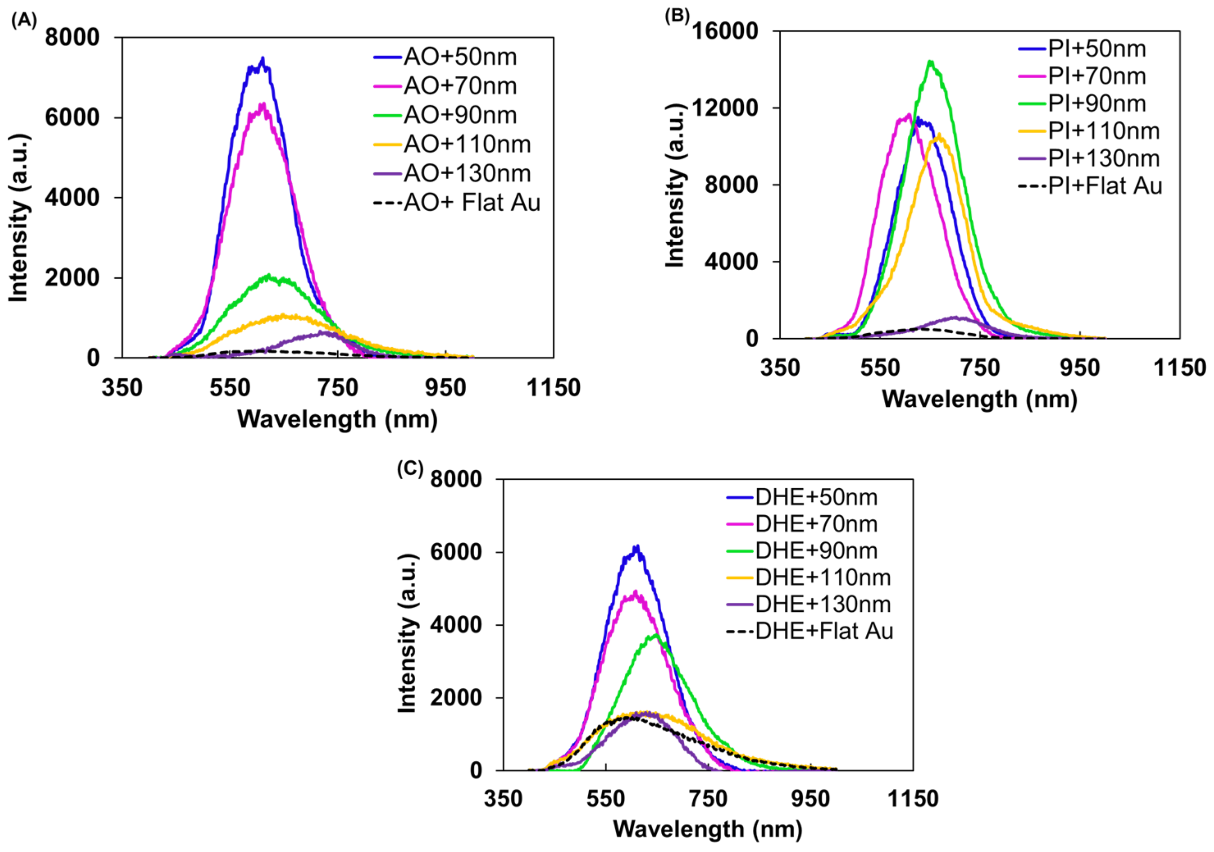

Fig. S4. Net reflectance intensity spectra of (a) AO (b) PI and (c) DHE in different nanoslit structures.

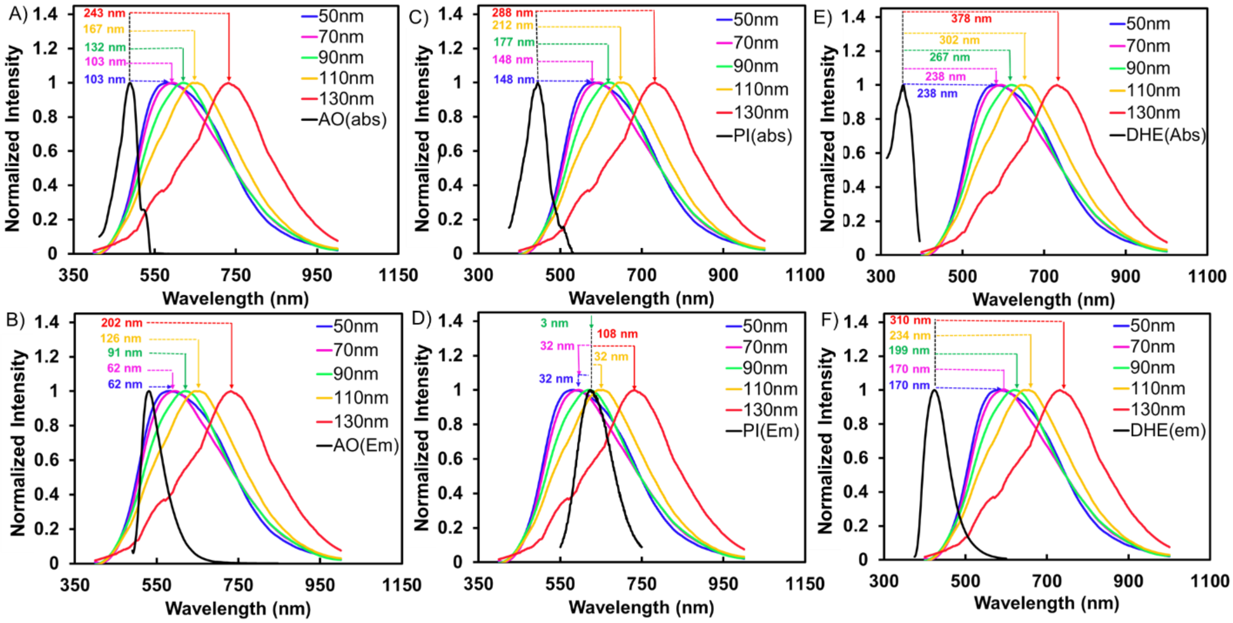

Fig S5. Normalized spectral overlap between the excitation and emission spectrum of AO, PI and DHE with reflection spectrum of nanoslits of widths 50 nm, 70 nm, 90 nm, 110 nm, and 130 nm respectively.

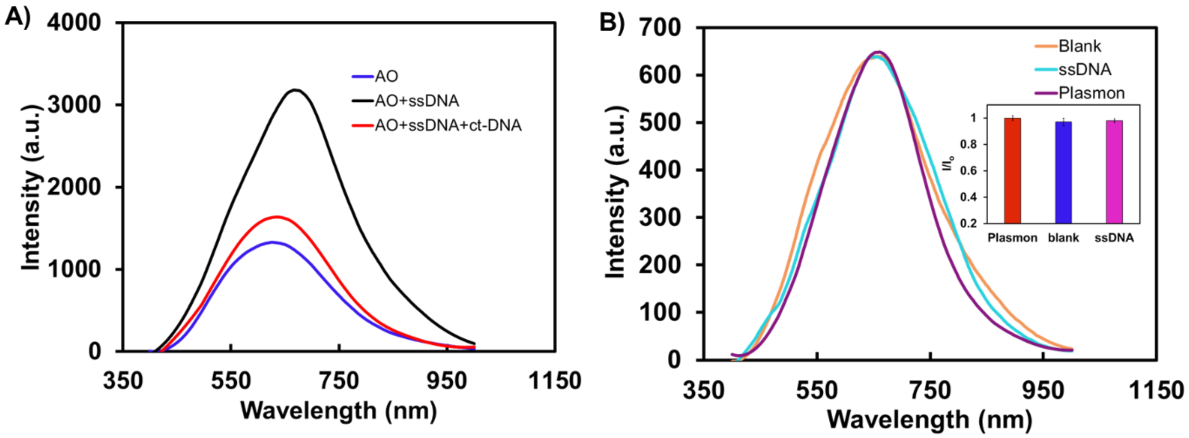

Fig. S6. (A) Cytoviva spectra of AO, AO and ssDNA probe in the presence and absence of ct-DNA at 110 nm nanoslit array; (B) The spectra of ssDNA directly in the nanoslits without a dye. The blank is a PBS buffer solution without ssDNA

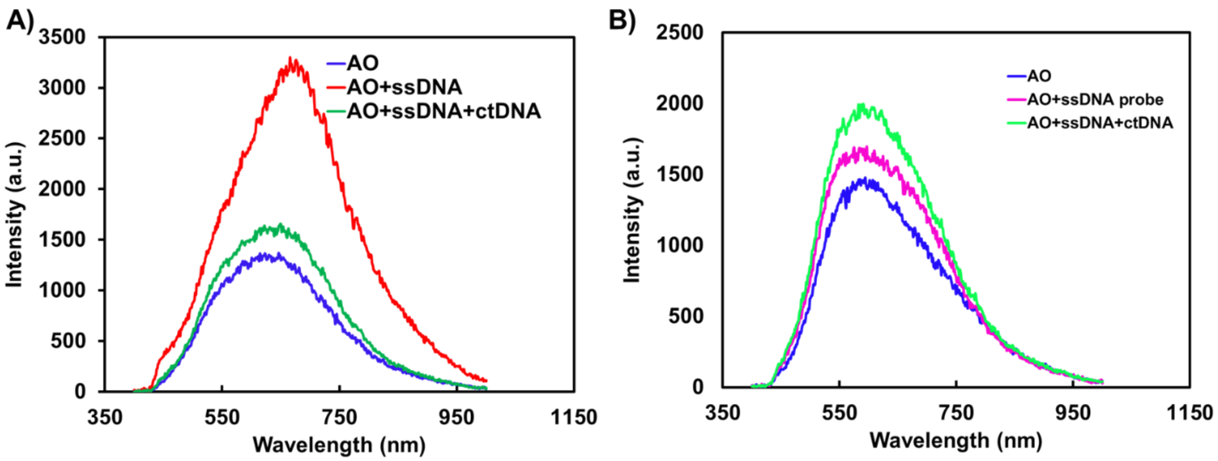

Fig S7. Effect of below-equilibrium concentration of acridine-ssDNA complex on intensity signal response. Ideally, the hybridization of ssDNA with its complementary strand in the presence of surface acridine is expected to cause signal intensity reduction (A) because acridine emits at a shorter wavelength (525 nm) in the presence of a double strand DNA. Since the emission spectrum of the dye is far away from plasmon resonance energy excited in the nanoslit, plasmon-exciton coupling efficiency is diminished and hence the quenching effect. However, figure (B) shows an enhancement in intensity by adding ctDNA to the surfaces where 4 μM of ssDNA probe was used in forming the ssDNA-AO substrate. This could be the result of a direct binding of ct-DNA to free acridine at the surface.

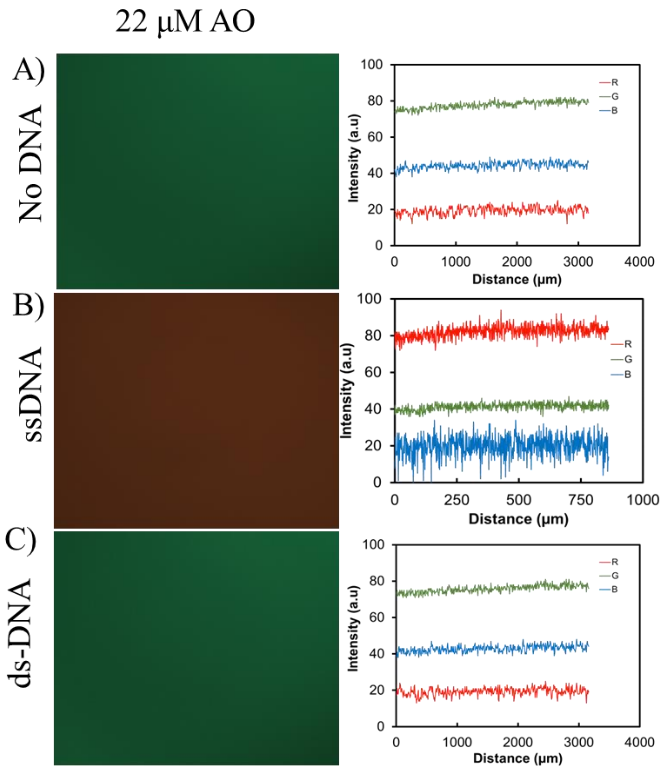

Fig. S8. Axio Z2M images and corresponding RGB intensity profile of (A). AO, (B) AO-ssDNA and (C) AO-dsDNA illuminated at 430nm.

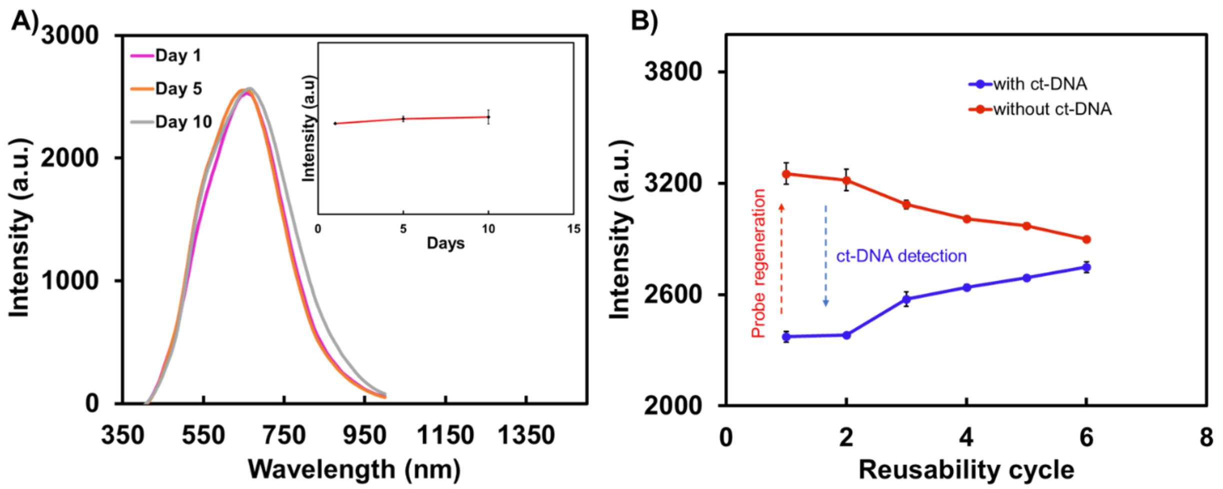

Fig. S9. A) The stability of sensor after 10 days storage. B) the reusability of the sensor after 6 regeneration cycles.

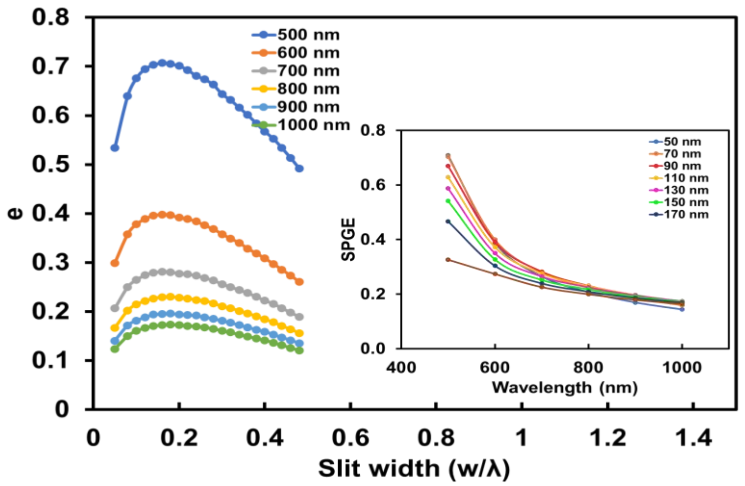

Fig. S10. Surface plasmon polariton generation efficiencies as a function of the slit width for different wavelength. Inset show the exponential decay of SPGE with increasing wavelength for different slit width.

Semi-analytical Modelling: Here, surface plasmon polariton efficiency taken to be the result of geometric diffraction and launching of bounded SPP modes on the flat interfaces surrounding the nanoslit. The signature of SPP-mode excitations was quantitatively recognized based on completeness theorem which offers useful information on the efficiency of SPP generation. For light propagating in a non-translational system, the transverse field pattern can be treated as a linear combination of forward- and backward-travelling bounded and radiative modes (Scheme S2).[1-3] Therefore, the SPP generation efficiency e of the transverse electromagnetic fields for our slit geometry and for $w/2 < x$ and $x < -w/2$ were estimated from equations 1-2. Note that $e_1 = e_2$ since in both cases one encounters Au/air interfaces, and the overall efficiency can be taken to be $e_1 + e_2$. Here we took account of the normalized SPP excitation strength $(|\alpha^+(x)|^2$ and $(|\alpha^+(x)|^2$ for $-w/2 < x < w/2$, since there is air-metal interface. Note that the field inside the slit consist of the downward and upward-reflected fundamental modes since we are dealing with light diffraction by slit arrays.

$$e_1 \; = \; |\alpha_1^+(w/2)|^2 = |\alpha_1^-(w/2)|^2 = \frac{4\,w'n_1^3}{\pi n_2^2}\,|\frac{\varepsilon^{1/2}}{\varepsilon + n_1^2}|\,|\frac{I_o}{1 + (n_1/n_2)w'I_o}|^2 \qquad (1)$$

$$e_2 \; = \; |\alpha_2^+(w/2)|^2 = |\alpha_2^-(w/2)|^2 = \frac{4\,w'n_1^3}{\pi n_1^2}\,|\frac{\varepsilon^{1/2}}{\varepsilon + n_2^2}|\,|\frac{I_o}{1 + (n_2/n_1)w'I_o}|^2 \qquad (2)$$

Where n_1, n_2, and n_3 are the refractive indices of the top Au-air and in-slit Au-air interfaces. Note that n_1= n_2=1. The normalized slit width is given by, w'=nw/λ where w is the slit width. The integrals I_1 and I_o were calculated numerically for different w' and ε for Au at different wavelengths.

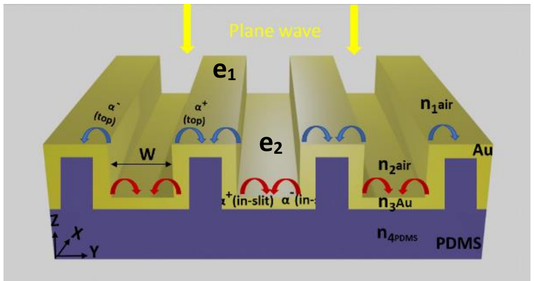

Scheme S2. The schematic illustrating the parameters for the nanoslit structure and surface plasmon generation under plane wave illumination at normal incidence

Equations (1 and 2) represent the semi-analytical expressions for SPP excitation efficiency when the slit channel is illuminated by fundamental slit mode. All plots were relative to the SPP excitation efficiency e_2 inside the slit. The efficiencies were obtained over a broad range of wavelength (500-1000nm).

Table S1a. Percentage decrease in intensity and intensity enhancement factor calculated for different nanoslit widths with respect to the 50 nm slit width. The intensity values used is shown in Table S1b. I_{50} and I_x are the intensities in W=50 nm and other slit width structures respectively.

**% Drop in intensity** $\mathbf{\Delta I(\%) = \frac{I_{50} - I_x}{(I_{50} + I_x)/2} * 100}$	**No dye**	**AO**	**PI**	**DHE**
**W (nm)**	ΔI_p	ΔI_{pd}(a.u)	ΔI_{pd}(a.u)	ΔI_{pd}(a.u)
**50**				
**70**	13%	16%	3%	31%
**90**	53%	102%	14%	141%
**110**	87%	137%	16%	160%
**130**	147%	165%	163%	184%

Table S1b: Measured scattering intensities on nanostructure area and plain Au surface with and without dye and the corresponding intensity enhancement factor

Plasmon and Control peak intensity	**No dye**		**With AO**		**With PI**		**With DHE**		**Intensity enhancement, I_E** $\mathbf{I_E = \frac{I_{pd} - I_p}{I_{cd} - I_c}}$		
W (nm)	I_p(a.u)	I_c(a.u)	I_{pd} (a.u)	I_{cd} (a.u)	I_{pd} (a.u)	I_{cd} (a.u)	I_{pd} (a.u)	I_{cd} (a.u)			
Control (plain Au)		43.02		171.62		512.79		2409.68	**AO**	**PI**	**DHE**
50	1645.25		8965.39		13098		6241.166		56.92	24.42	1.94
70	1445.19		7651.33		12777.00		4588.16		48.25	24.12	1.32
90	946.90		2930.92		15044.31		1081.05		15.43	30.01	0.06
110	644.49		1669.58		11113.39		692.71		7.97	22.28	0.02
130	253.05		861.09		1327.56		267.48		4.72	2.29	0.01

Ip = intensity of nanostructure without dye; Ic= intensity of plain Au surface without dye

Ipd = intensity of nanostructure with dye; Icd=intensity of plain Au with dye

I_E=reflectance intensity enhancement factor

Table S2. Sensitivity of existing biosensors for SARS-CoV-2 testing

**Methods**	**Transducer**	**Target**	**Detection limit**	**Detection time**

ELISA[4-5]		SARS-CoV-2 IgG in serum	1.953 ng/mL	5h
Photoluminescence spectroscopy[6]	Semiconductor polymer	SARS-CoV-2 IgG in serum	0.0125 μg/mL	
SPR[7]	Gold film	S protein	0.08 pg/mL	<1 min
Fluorescence[8]	Fluorescent probe	SARS-CoV-2 E & N gene	10 copies/μL	30-40 min
colorimetric[9]	fluorescein dye	SARS-CoV-2	12 copies/ reaction	60 min
Colorimetric/SPR[10]	QD/Au Npcs	SARS-CoV-2 N-gene RNA	0.18 ng/ μL	10min
EFT[11]	Graphene	SARS-CoV-2 in PBS	1 fg/mL	<2 min
Electrochemical[12]	Electrode	SARS-CoV-2 N & S genes	1 copy/ μL	<2 hours
Electrochemical[13]	electrodes	SARS-CoV-2 antibody in saliva	2.3 RNA copies/ μL	50 min
SPR[14]	Au nanocup array	SARS-CoV-2 antigen in saliva	370 vp/mL	15 min
SPR[15]	Au nanoislands	SARS-CoV-2 specific sequence	0.22 pM	
This work	Au nanoslit array	SARS-CoV-2 specific sequence	0.21 nM	25 min

Table S3. Numerical data of I_0 and I_1 for different values of w' for gold at different wavelengths. Note that I_0 is same for Au at varying wavelengths

		400 nm ε=-1.66 + 5.74j	500 nm ε=-2.5676 + 3.6391j	600 nm ε=-9.3875 + 1.5292j	700 nm ε=-16.486 + 1.0643j	800 nm ε=-24.061 + 1.5068j	900 nm ε=-32.719 + 1.9955j	1000 nm ε=-41.849 + 2.9477j
w'	I_o	I_1	I_1	I_1	I_1	I_1	I_1	I_1
0.05	3.13-5.52j	-0.44-3.53j	-0.68-3.33j	-0.20-2.99j	-0.02-3.02j	0.07-3.05j	0.13-3.06j	0.17-3.07j
0.08	3.11-4.54j	-0.17-3.50j	-0.41-3.30j	0.07-2.93j	0.25-2.96j	0.34-2.98j	0.40-3.00j	0.44-3.00j
0.1	3.09-4.09j	0.01-3.46j	-0.24-3.27j	0.23-2.88j	0.42-2.90j	0.51-2.92j	0.57-2.93j	0.62-2.94j
0.12	3.07-3.73j	0.19-3.41j	-0.07-3.23j	0.39-2.82j	0.58-2.84j	0.67-2.85j	0.74-2.86j	0.78-2.87j
0.14	3.04-3.37j	0.36-3.35j	0.10-3.17j	0.55-2.75j	0.74-2.76j	0.83-2.77j	0.90-2.78j	0.95-2.78j
0.16	3.01-3.07j	0.53-3.28j	0.26-3.11j	0.69-2.67j	0.88-2.67j	0.98-2.68j	1.05-2.68j	1.10-2.68j
0.18	2.98-2.81j	0.69-3.21j	0.41-3.04j	0.83-2.58j	1.02-2.57j	1.12-2.58j	1.19-2.58j	1.24-2.58j
0.2	2.94-2.61j	0.84-3.12j	0.55-2.97j	0.96-2.48j	1.15-2.46j	1.25-2.47j	1.32-2.46j	1.37-2.46j
0.22	2.90-2.39j	0.99-3.02j	0.69-2.88j	1.08-2.38j	1.27-2.35j	1.37-2.35j	1.44-2.34j	1.49-2.33j
0.24	2.86-2.18j	1.13-2.91j	0.82-2.78j	1.19-2.27j	1.38-2.23j	1.48-2.22j	1.55-2.21j	1.60-2.20j
0.26	2.81-1.99j	1.26-2.80j	0.95-2.68j	1.28-2.15j	1.47-2.10j	1.58-2.09j	1.64-2.07j	1.69-2.06j
0.28	2.77-1.82j	1.37-2.68j	1.06-2.58j	1.37-2.03j	1.56-1.97j	1.66-1.95j	1.73-1.93j	1.78-1.92j
0.3	2.72-1.69j	1.48-2.55j	1.16-2.46j	1.45-1.90j	1.63-1.83j	1.73-1.80j	1.80-1.78j	1.84-1.77j
0.32	2.66-1.55j	0.99-3.02j	1.26-2.34j	1.51-1.77j	1.69-1.69j	1.79-1.66j	1.86-1.63j	1.90-1.62j
0.34	2.61-1.41j	1.67-2.28j	1.34-2.22j	1.57-1.64j	1.74-1.55j	1.84-1.51j	1.90-1.48j	1.94-1.46j
0.36	2.55-1.29j	1.74-2.13j	1.42-2.09j	1.61-1.50j	1.78-1.41j	1.87-1.37j	1.93-1.33j	1.97-1.31j
0.38	2.49-1.17j	1.80-1.99j	1.48-1.96j	1.64-1.37j	1.80-1.27j	1.89-1.22j	1.95-1.18j	1.99-1.16j
0.4	2.43-1.05j	1.85-1.84j	1.53-1.83j	1.66-1.24j	1.81-1.13j	1.90-1.07j	1.96-1.04j	2.00-1.01j
0.42	2.37-0.95j	1.90-1.69j	1.58-1.70j	1.67-1.11j	1.82-0.99j	1.90-0.93j	1.95-0.89j	1.99-0.86j
0.44	2.31-0.86j	1.92-1.55j	1.61-1.57j	1.67-0.98j	1.81-0.85j	1.88-0.79j	1.93-0.75j	1.97-0.72j
0.46	2.25-0.78j	1.94-1.40j	1.63-1.44j	1.66-0.86j	1.79-0.72j	1.86-0.66j	1.91-0.61j	1.94-0.58j
0.48	2.19-0.71j	1.94-1.26j	1.64-1.31j	1.64-0.74j	1.76-0.60j	1.82-0.53j	1.87-0.48j	1.90-0.45j

Table S4. Surface plasmon generation efficiency calculation under different incident wavelengths for different nanoslit

w	500 nm	**600 nm**	**700 nm**	**800 nm**	**900 nm**	**1000 nm**
50	0.709	0.389	0.264	0.206	0.169	0.143
70	0.704	0.400	0.281	0.224	0.186	0.159
90	0.670	0.391	0.282	0.231	0.194	0.169
110	0.628	0.372	0.276	0.230	0.197	0.173
130	0.588	0.350	0.265	0.225	0.195	0.174
150	0.542	0.327	0.252	0.218	0.191	0.172
170	0.467	0.304	0.239	0.209	0.186	0.169
190	0.327	0.274	0.226	0.200	0.179	0.164
210	0.068	0.231	0.210	0.190	0.172	0.159

References

(1) Bagra, B.; Zhang, W.; Zeng, Z.; Mabe, T.; Wei, J. Plasmon-Enhanced Fluorescence of Carbon Nanodots in Gold Nanoslit Cavities. *Langmuir* **2019,** *35* (27), 8903-8909, DOI: 10.1021/acs.langmuir.9b00448.

(2) Zeng, Z.; Mendis, M. N.; Waldeck, D. H.; Wei, J. A semi-analytical decomposition analysis of surface plasmon generation and the optimal nanoledge plasmonic device. *RSC Adv.* **2016,** *6* (21), 17196-17203, DOI: 10.1039/C6RA01105E.

(3) Lalanne, P.; Hugonin, J. P.; Rodier, J. C. Approximate model for surface-plasmon generation at slit apertures. *J. Opt. Soc. Am. A* **2006,** *23* (7), 1608-1615, DOI: 10.1364/JOSAA.23.001608.

(4) Vernet, R.; Charrier, E.; Grogg, J.; Mach, N. A Quantitative ELISA Protocol for Detection of Specific Human IgG against the SARS-CoV-2 Spike Protein. *Vaccines* **2021,** *9* (7), 770, DOI: 10.3390/vaccines9070770.

(5) Eftekhari, A.; Alipour, M.; Chodari, L.; Maleki Dizaj, S.; Ardalan, M.; Samiei, M.; Sharifi, S.; Zununi Vahed, S.; Huseynova, I.; Khalilov, R.; Ahmadian, E.; Cucchiarini, M. A Comprehensive Review of Detection Methods for SARS-CoV-2. *Microorganisms* **2021,** *9* (2), 232, DOI: 10.3390/microorganisms9020232.

(6) Bassi, M. D.; Araujo Todo Bom, M.; Terribile Budel, M. L.; Maltempi de Souza, E.; Müller dos Santos, M.; Roman, L. S. Optical Biosensor for the Detection of Infectious Diseases Using the Copolymer F8T2 with Application to COVID-19. *Sensors* **2022,** *22* (15), 5673, DOI: 10.3390/s22155673.

(7) Dai, Z.; Xu, X.; Wang, Y.; Li, M.; Zhou, K.; Zhang, L.; Tan, Y. Surface plasmon resonance biosensor with laser heterodyne feedback for highly-sensitive and rapid detection of COVID-19 spike antigen. *Biosens. Bioelectron.* **2022,** *206,* 114163, DOI: 10.1016/j.bios.2022.114163.

(8) Broughton, J. P.; Deng, X.; Yu, G.; Fasching, C. L.; Servellita, V.; Singh, J.; Miao, X.; Streithorst, J. A.; Granados, A.; Sotomayor-Gonzalez, A.; Zorn, K.; Gopez, A.; Hsu, E.; Gu, W.; Miller, S.; Pan, C.-Y.; Guevara, H.; Wadford, D. A.; Chen, J. S.; Chiu, C. Y. CRISPR–Cas12-based detection of SARS-CoV-2. *Nat. Biotechnol.* **2020,** *38* (7), 870-874, DOI: 10.1038/s41587-020-0513-4.

(9) Zhu, X.; Wang, X.; Han, L.; Chen, T.; Wang, L.; Li, H.; Li, S.; He, L.; Fu, X.; Chen, S.; Xing, M.; Chen, H.; Wang, Y. Multiplex reverse transcription loop-mediated isothermal

amplification combined with nanoparticle-based lateral flow biosensor for the diagnosis of COVID-19. *Biosens. Bioelectron.* **2020,** *166*, 112437, DOI: 10.1016/j.bios.2020.112437.
(10) Moitra, P.; Alafeef, M.; Dighe, K.; Frieman, M. B.; Pan, D. Selective Naked-Eye Detection of SARS-CoV-2 Mediated by N Gene Targeted Antisense Oligonucleotide Capped Plasmonic Nanoparticles. *ACS Nano* **2020,** *14* (6), 7617-7627, DOI: 10.1021/acsnano.0c03822.
(11) Seo, G.; Lee, G.; Kim, M. J.; Baek, S.-H.; Choi, M.; Ku, K. B.; Lee, C.-S.; Jun, S.; Park, D.; Kim, H. G.; Kim, S.-J.; Lee, J.-O.; Kim, B. T.; Park, E. C.; Kim, S. I. Rapid Detection of COVID-19 Causative Virus (SARS-CoV-2) in Human Nasopharyngeal Swab Specimens Using Field-Effect Transistor-Based Biosensor. *ACS Nano* **2020,** *14* (4), 5135-5142, DOI: 10.1021/acsnano.0c02823.
(12) Chaibun, T.; Puenpa, J.; Ngamdee, T.; Boonapatcharoen, N.; Athamanolap, P.; O'Mullane, A. P.; Vongpunsawad, S.; Poovorawan, Y.; Lee, S. Y.; Lertanantawong, B. Rapid electrochemical detection of coronavirus SARS-CoV-2. *Nat. Commun.* **2021,** *12* (1), 802, DOI: 10.1038/s41467-021-21121-7.
(13) Najjar, D.; Rainbow, J.; Sharma Timilsina, S.; Jolly, P.; de Puig, H.; Yafia, M.; Durr, N.; Sallum, H.; Alter, G.; Li, J. Z.; Yu, X. G.; Walt, D. R.; Paradiso, J. A.; Estrela, P.; Collins, J. J.; Ingber, D. E. A lab-on-a-chip for the concurrent electrochemical detection of SARS-CoV-2 RNA and anti-SARS-CoV-2 antibodies in saliva and plasma. *Nature Biomedical Engineering* **2022,** *6* (8), 968-978, DOI: 10.1038/s41551-022-00919-w.
(14) Huang, L.; Ding, L.; Zhou, J.; Chen, S.; Chen, F.; Zhao, C.; Xu, J.; Hu, W.; Ji, J.; Xu, H.; Liu, G. L. One-step rapid quantification of SARS-CoV-2 virus particles via low-cost nanoplasmonic sensors in generic microplate reader and point-of-care device. *Biosens. Bioelectron.* **2021,** *171*, 112685, DOI: 10.1016/j.bios.2020.112685.
(15) Qiu, G.; Gai, Z.; Tao, Y.; Schmitt, J.; Kullak-Ublick, G. A.; Wang, J. Dual-Functional Plasmonic Photothermal Biosensors for Highly Accurate Severe Acute Respiratory Syndrome Coronavirus 2 Detection. *ACS Nano* **2020,** *14* (5), 5268-5277, DOI: 10.1021/acsnano.0c02439.
